# Prosocial and Aggressive Behavior: A Longitudinal Study

**DOI:** 10.1111/mono.12427

**Published:** 2021-05-11

**Authors:** Dale F. Hay, Amy L. Paine, Oliver Perra, Kaye V. Cook, Salim Hashmi, Charlotte Robinson, Victoria Kairis, Rhiannon Slade

**Affiliations:** ^1^ School of Psychology Cardiff University; ^2^ School of Nursing and Midwifery, Centre for Evidence and Social Innovation Queen's University Belfast; ^3^ Department of Psychology Gordon College; ^4^ Department of Psychology, Institute of Psychiatry, Psychology and Neuroscience King's College London

## Abstract

Developmental theorists have made strong claims about the fundamental prosocial or aggressive nature of the human infant. However, only rarely have prosocial behavior and aggression been studied together in the same sample. We charted the parallel development of both behaviors from infancy to childhood in a British community sample, using a two‐construct, multimethod longitudinal design.

Data were drawn from the Cardiff Child Development Study (CCDS), a prospective longitudinal study of a volunteer sample of parents and their firstborn children. A sample of 332 mothers was recruited from National Health Service (NHS) prenatal clinics and general practice clinics in Wales, UK, between Fall of 2005 and Summer of 2007. Potential participants represented the full range of sociodemographic classifications of neighborhoods. Participating families were divided about equally between middle‐ and working‐class families, were somewhat more likely to have sons than daughters, and the majority (90%) were in a stable partnership. In response to standard categories recommended for use in Wales at the time, the majority (93%) of mothers reported themselves as Welsh, Scottish, English, or Irish; most others named a European or South Asian nationality.

Of the 332 families agreeing to participate, 321 mothers (*M*_age_ = 28 years) and 285 partners (*M*_age_ = 31 years) were interviewed during the pregnancy and 321 of the families contributed data at least once after the child's birth. After an initial home visit at 6 months, data collection occurred in four additional waves of testing when children's mean ages were approximately 1, 1.5, 2.5, and 7 years. Data collection alternated between family homes and Cardiff University. Of those families seen after the child's birth, 89% were assessed at the final wave of testing. Data collection ended in 2015.

Methods included direct observation, experimental tasks, and collection of reports from mothers, fathers, other relatives or family friends, and classroom teachers. Interactions with a familiar peer were observed at 1.5 years. Interactions with unfamiliar peers took place during experimental birthday parties at 1 and 2.5 years. At 7 years, parents were interviewed, parents and teachers completed questionnaires, and the children engaged in cognitive and social decision‐making tasks.

Based on reports from parents and other informants who knew the children well, individual differences in both prosocial behavior and aggression were evident in children. Both types of behavior showed stability across the second and third years. The association between prosocial behavior and aggression changed over time: at 1.5 years, they were not significantly related (the association approached zero), but they became negatively correlated by 3 years.

Different patterns were seen when children played with familiar versus unfamiliar peers. At 1.5 years, when children were observed at home with a familiar peer, prosocial behavior and aggression were unrelated, thus showing a pattern of results like that seen in the analysis of informants' reports. However, a different pattern emerged during the experimental birthday parties with unfamiliar peers: prosocial behavior and aggression were positively correlated at both 1 and 2.5 years, contributing to a general sociability factor at both ages.

Gender differences in prosocial behavior were evident in informants' reports and were also evident at the 1‐year (though not the 2.5‐year) birthday parties. In contrast, gender differences in both prosocial behavior and aggression were evident by 7 years, both in children's aggressive decision‐making and in their parents' and teachers' reports of children's aggressive behavior at home and school.

By age 7, children's aggressive decision‐making and behavior were inversely associated with their verbal skills, working memory, and emotional understanding. Some children had developed aggressive behavioral problems and callous‐unemotional traits. A few (12%) met diagnostic criteria for conduct disorder or oppositional‐defiant disorders, which had been predicted by early angry aggressiveness and lack of empathy for other people.

Taken together, the findings revealed a gradual disaggregation of two ways in which children interact with other people. Individual differences in both prosocial behavior and aggression revealed continuity over time, with gender differences emerging first in prosocial behavior, then in aggression.

Restrictions in the participant sample and the catchment area (e.g., all were first‐time parents; all were drawn from a single region in the United Kingdom) mean that it is not possible to generalize findings broadly. It will be important to expand the study of prosocial behavior and aggression in other family and environmental contexts in future work. Learning more about early appearing individual differences in children's approaches to the social world may be useful for both educational and clinical practice.

## I Introduction

In this monograph, we explore the parallel development of aggression and prosocial behavior from infancy to middle childhood, with a particular focus on individual differences in both behaviors. One of the most distinctive traits of the human species is a capacity for social engagement with other people, which can encompass both cooperation and conflict, prosocial behavior, and violence. Early forms of prosocial behavior and aggression emerge in the first 2 years. However, they are only rarely studied in relation to each other, so little is known about their relative rates of occurrence and how they relate to each other. In the first 3 years, are prosocial behavior and aggression already negatively correlated? Positively correlated? Or unrelated?

If there are individual differences in early life, are they meaningful? It is not yet clear whether very young children's prosocial and forceful acts predict later prosocial behavior and aggression. Put the other way round, do later differences among individuals originate in the first years of life?

To provide information about the development of both behaviors, we present longitudinal findings from a community sample in which both constructs were reported on by multiple informants, directly observed during peer interaction, and assessed during experimental tasks. We asked the following questions: Do individual differences in prosocial behavior and aggression emerge early and persist over time? Do young children who share, help, and cooperate possess better prosocial skills in later childhood, at school as well as home? Are those young children who express anger and use force at high rates more likely to have aggressive behavioral problems later on? And how do these two forms of social behavior relate to each other over time?

Our attempt to answer these questions builds upon the separate developmental literatures on aggression and prosocial behavior. We review these literatures below. First, however, we present our working definitions of aggression and prosocial behavior.

## Definitions

### Aggression

In the work reported in this monograph, we focused on physical aggression, that is, the use of physical force against another person's body, as opposed to verbal or social aggression. We see children's physically aggressive acts as intentional motor actions, as opposed to accidental actions such as bumping into someone by mistake. However, we do not require evidence that the child intended to harm the other person because proof of intent would be hard or perhaps impossible to establish in this age range. Rather, children's physical aggression is operationally defined as forceful hitting, biting, pushing, or pulling on another person's body.

### Prosocial Behavior

In general, we define prosocial behavior as social actions that provide other people with resources, instrumental help, comfort, or the expression of empathic/sympathetic feelings. In the age range studied, we focus on early forms of sharing, cooperation, helping, and comforting. We do not require evidence that the children have selfless motives, and so we do not assume that their prosocial actions qualify as forms of altruism. However, parents’ self‐reports of prosocial behavior included altruistic activities such as donations to charity.

## Research on Children's Early Aggression

### Theoretical Perspectives

The developmental literature has long focused on individual differences in aggression and the underlying processes that promote it. In the 20th century, much research on children's aggression was conducted within the social learning tradition. Experimenters tested for the effects of both positive and negative reinforcement of aggression, and for observational learning from aggressive role models (for reviews of this work, see Bandura & Walters, 1963; Cairns, 1979). The social learning theorists identified paradoxical effects of the social environment on children's aggression; for example, when parents punished aggression, they simultaneously provided models of the use of force, which might induce, rather than inhibit, the children's own aggression (Patterson & Cobb, 1971). Social learning theorists drew attention to the complexities of family interactions and therefore had an impact on family systems theory and clinical practice (Granic et al., 2003).

Subsequent studies of social influences on aggression extended their focus beyond social reinforcement and observational learning. Some investigators drew attention to early parent–infant interaction and the development of insecure attachments as contributors to the development of aggression (e.g., Erickson et al., 1985; Martin, 1981). Others focused on parents’ strategies for managing young children's anger and aggressive impulses (e.g., Belsky et al., 1996), rather than on the way in which children learned how to be aggressive in the first place.

In the 21st century, these more nuanced investigations of social influences on aggression have been undertaken in parallel with a resurgence of interest in the biological underpinnings of aggressive behavior and a focus on the earliest years of life (e.g., Moffitt, 2005; Tremblay, 2000). In some cases, the new work on early signs of aggression was a rebuttal to traditional social learning theory. For example, Tremblay has claimed that, as infants’ motor skills evolve, infants naturally and inevitably deploy physical aggression against others. He argued that early aggression should be seen as a normative developmental milestone:

physical aggression is not a behavior children learn, like reading or writing, nor an illness children “catch,” like poliomyelitis or smallpox. It is rather a behavior like crying, eating, sleeping, grasping, throwing, and running, which young humans exhibit when the physiological structure is in place, but then learn to control with experience *(Tremblay & Nagin, 2005, p. 95)*.

Within this perspective, social learning still plays a role. Tremblay proposed that what young children learned was not to be aggressive per se—aggressive actions would emerge naturally—but rather they acquired the skills needed to control their natural aggressive tendencies (Tremblay, 2000, 2010). Tremblay has referred ironically to this formulation as the “original sin hypothesis” and noted that his theoretical views draw upon the philosophical stance taken by Hobbes as opposed to Rousseau (Tremblay, 2010).

Tremblay and his colleagues tested these ideas by studying infants and very young children in large, representative community samples. These included the Québec Longitudinal Study (e.g., Baillargeon et al., 2007; Côté et al., 2006), which revealed that signs of physical aggression were evident by the second year. Toddlers’ use of aggression has also been recorded in other large, representative samples (e.g., Alink et al., 2008; NICHD Early Child Care Research Network, 2004) and high‐risk groups (e.g., Lorber et al., 2018).

There are debates about the age at which children's behavior can be described as aggressive, and theorists disagree about definitions. Some developmental psychologists have argued that, to be defined as aggressive, the child's use of physical force has to be intentional (e.g., Parke & Slaby, 1983), while other theorists recommend defining aggression operationally as acts of physical force, without requiring intentionality, which makes it easier to detect aggression in very young children and nonhuman species (e.g., Gendreau & Archer, 2005). Thus, any estimates of the age of onset of aggression depend on the definitions used and the extent to which efforts are made to measure the child's intentions. Studies in the social learning tradition focused on experimental challenges that might evoke aggression, such as the famous Bobo Doll experiments (e.g., Bandura et al., 1961). The studies of larger community samples typically included examples of aggression (e.g., “hits”) reported by parents.

When interpreting findings about children's aggression, it is important to distinguish between competence and performance. The motor skills required to hit or kick other people develop in the first 2 years of life; but how often do toddlers show these behaviors? What factors promote or reduce their occurrence? Do early individual differences in aggressive tendencies endure over time?

### Research Topics

Across different theoretical traditions and methodologies, some common questions about the development of aggression have been raised, as reviewed briefly below.

#### What Are the Different Dimensions of Early Aggression?

Legal frameworks make distinctions between intentional and accidental, justified (e.g., acts of self‐defense) and unjustified acts of violence. Similarly, developmental scientists have long made distinctions between different types of aggression. For example, Hartup (1974) distinguished between hostile aggression (i.e., forceful acts undertaken for their own sake) and instrumental aggression (i.e., the use of force to obtain a goal). Distinctions are also made between proactive aggression (i.e., forceful acts that begin interactions) versus reactive aggression that includes forceful responses to provocations from others (e.g., Dodge & Coie, 1987; Séguin & Zelazo, 2004).

Such distinctions can be discerned in very young children's interactions with siblings and peers. For example, young children are significantly more likely to use force instrumentally by tugging on peers’ toys than by hitting or kicking other people for no apparent reason (Hay & Ross, 1982; Hay et al., 2011c). Force is often used reactivel*y*, in response to provocations from other toddlers, rather than as the first act in a social encounter (Hay et al., 2011a).

When attempting to study the different forms of early aggression, methodological choices become important. One important issue is how to synthesize the data provided via questionnaires, interviews, direct observation, and experimental tasks. Questionnaire items typically measure whether a child sometimes or often hits or kicks or expresses anger (e.g., Tremblay et al., 1999), not the context in which such behaviors are shown. Thus, it is not always possible to determine from informants’ reports whether the aggression being reported is intentional or accidental, hostile or instrumental, proactive or reactive in nature. Despite this limitation, parents’ and teachers’ reports do draw on observations of familiar children over a long period of time. In contrast, the evidence from short‐term experiments and observational studies records children's reactions to a particular social situation on a given day, not their long‐term aggressive tendencies. Different studies use different methods, which makes comparing across samples difficult.

#### Competence Versus Performance

Several studies suggest that physically aggressive behaviors emerge by the second year of life (e.g., Alink et al., 2006; Côté et al., 2006; Hay et al., 2011c). However, most children do not use their aggressive motor skills at high rates. Instrumental aggression (e.g., tugging on toys) is more common than the use of bodily force. For example, in a survey of 572 families in the province of Québec (Tremblay et al., 1999), parents reported that 70% of 17‐month‐olds took objects away from other people, but only 18% did so often. Similarly, 15% of them were reported to hit other people, but fewer than 1% did so often.

Similarly, in a combined data set of five observational studies (*N* = 323) that used the same coding system (Hay et al., 2011c), over 70% of toddlers tugged on their peers’ toys at least once, at a mean rate of 3.3 acts per hour. Only 50% hit or pushed other children, at a mean rate of 1.6 acts per hour (Hay et al., 2011c). Thus, both informants’ reports and direct observation reveal individual differences in early aggression.

#### Biological Risk Factors

The renewed interest in the biological underpinnings of aggression has led to different strands of research on genetic risk factors (D'Onofrio et al., 2003; Dick et al., 2006; Moffitt, 2005; Porsch et al., 2016; Rhee & Waldman, 2002) and neurobiological correlates (Séguin et al., 2004; Van Goozen et al., 2007). Developmental theorists have sought evidence for the interactive effects of genetic factors and the family environment (e.g., Caspi et al., 2005). New technologies have been used to identify genetic contributions to aggression, including genome‐wide association studies (e.g., Dick et al., 2011).

Children's persistent aggression and related conduct problems have sometimes been seen as neurobiological disorders (e.g., Van Goozen et al., 2007). The focus in this literature has been on deficits in emotion processing and self‐regulation that make it difficult for children to control their aggressive impulses. In some cases, this is linked to the development of callous‐unemotional (CU) traits that limit empathy and foster antisocial behavior (e.g., Frick et al., 2003). Early‐onset aggression coupled with neurobiological problems is a key predictor of conduct disorders (CDs) that persist into later childhood (e.g., Brennan & Shaw, 2013; Broidy et al., 2003).

#### Parent–Child Interaction

Parents’ own history of antisocial behavior may put children at risk for aggression, not just because of genetic transmission but the family environments parents construct. Within the social learning perspective, Patterson (1982) created coercion theory, which proposes a developmental pathway from conflict‐ridden family environments to children's later aggression. Parents with a history of a difficult upbringing and current financial problems often find the transition to parenthood challenging, particularly if both the parents and their children have similar problems in anger and self‐regulation (van Goozen et al., 1997). For example, in an urban British sample, mothers with a history of antisocial behavior were more likely than other women to become depressed in pregnancy; both maternal variables predicted children's later violence (Hay et al., 2010).

Parents’ negativity and harsh punishment increases the likelihood that children will become aggressive. For example, in a sample of children at risk for behavioral problems, the mother's expression of negative emotion and harsh punishment was associated with her children's noncompliance and later behavioral problems (e.g., Combs‐Ronto et al., 2009). However, the absence of harsh punishment is not in itself sufficient; for example, in a Chinese sample, both harsh and overly indulgent parenting predicted children's later aggression (Xu et al., 2009). Nevertheless, positive parenting reduces a child's risk for aggressive behavior (Perra et al., in press; Waller et al., 2018). Interventions that foster positive engagement between parent and child reduce coercive family interactions, which in turn lowers the child's risk for behavioral problems (Sitnick et al., 2015).

#### Gender

Gender differences in levels of aggression, which reflect biological factors as well as social experiences that differentiate boys and girls (Keenan & Shaw, 1997), are not immediately evident; rather, girls’ and boys’ aggression levels begin to differ around the second birthday (Baillargeon et al., 2007; Crockenberg et al., 2008; Hay et al., 2011c). These emerging gender differences are predated by earlier differences in negative emotionality, linked to girls’ and boys’ prenatal experiences (Braithwaite et al., 2017). Girls’ more rapid rate of maturation may possibly foster communication skills and self‐regulation, which would then help control their aggressive tendencies.

#### Continuity and Change Over Time

Longitudinal studies have revealed both change and continuity in aggression, identifying different patterns over time from the second year of life onwards (e.g., Côté et al., 2006; NICHD Early Child Care Research Network, 2004; Perra et al., in press; Tremblay et al., 2005). Taken together, these studies show that there is no single pattern of decline in aggressiveness from infancy to later childhood. Rather, some children never show aggression at high rates; others do; and still others show different patterns of change over time. However, we know relatively little about the interplay between these trends in aggressiveness and the emergence of prosocial behavior.

## Early Prosocial Behavior

### Theoretical Perspectives

The study of prosocial development has been undertaken with reference to broader theories of altruism. Moral philosophers (Nagel, 1970), evolutionary biologists (e.g., de Waal, 2008; Krebs & van Hesteren, 1994; Trivers, 1971), and social psychologists (e.g., Cialdini et al., 1987; Darley & Latané, 1968) have long discussed the concept of selfless altruism, whereby one individual engages in self‐sacrificing acts for the benefit of another. They speculated about the biological and social processes that underpin altruism such as reciprocity (Trivers, 1971) and empathic motivation (de Waal, 2008; Krebs & van Hesteren, 1994).

Developmental psychologists began to focus on a broader category of behaviors, not all of which would qualify as self‐sacrificing altruism but could be considered *prosocial* (Wispé, 1972). In this monograph, we follow Marian Radke‐Yarrow's definition of prosocial behaviors as those that “aid or benefit another person” (Radke‐Yarrow et al., 1983). They include sharing, cooperating, helping, and ministering to someone's distress. In the observations of early prosocial behavior reported in subsequent chapters, we did not require the children to be self‐sacrificing. We note, however, that some contemporary theorists argue that these early prosocial actions as steps on the developmental pathway to human altruism (Dahl & Paulus, 2019).

In the 20th century, the study of children's prosocial behavior drew upon findings from social psychological studies of bystander intervention (e.g., Darley & Latané, 1968) and of cooperative and competitive decision‐making (e.g., Deutsch, 2014). Theories of stages of moral understanding remain influential (Hoffman, 1979; Malti & Noam, 2016; Miller et al., 1996). However, children's prosocial behavior was also studied within the framework of social learning theory (e.g., Doescher & Sugawara, 1992). Modeling by parents and in the media do influence children's prosocial behavior (e.g., Huesmann & Eron, 1986; Radke‐Yarrow & Zahn‐Waxler, 1986). More recent work has drawn attention to the ways in which parents foster and scaffold early prosocial behavior (e.g., Brownell, 2016). However, paradoxical findings have emerged. Parents and teachers may fail to reinforce children's attempts to share or comfort others and may indeed discourage those behaviors (Caplan, 1993; Caplan & Hay, 1989; Eisenberg, 1988). More recently, an evolutionary framework has been applied to the study of early prosocial behavior, with a focus on empathy and helping.

#### Theories of Empathy

Both psychoanalysis and evolutionary theory drew attention to the origins of empathy, particularly infants’ crying in response to other people's distress (e.g., Sagi & Hoffman, 1976). Psychoanalysts argued that infants experienced a blurred boundary between themselves and others (Freud, 1949; Winnicott, 1960). In this view, infants may cry when another person cries only because they cannot tell the difference between their own distress and that of others.

In contrast, de Waal (2008) proposed an evolutionary account of empathy that rests on the detection of emotion, not confusion between self and other. He identified three levels of empathy: *emotional contagion*, *sympathetic concern*, and *empathic perspective‐taking*, and argued that the first two could be discerned in nonhuman species such as the great apes. Infants may similarly show emotional contagion, smiling when another smiles (Field et al., 1985), crying when another cries (Sagi & Hoffman, 1976). Experiments demonstrated that infants can indeed distinguish between their own previously recorded cries and the cries of another infant (Dondi et al., 1999). They can tell the difference between videos of themselves and other infants (Legerstee et al., 1998). Thus, crying in response to another infant's cry is now seen as an early step on the pathway toward empathy and compassion (Davidov et al., 2013). Infants’ concern for people in distress can be detected by 3 months of age (Davidov et al., 2020).

Furthermore, as they grow older, infants show signs of what de Waal called *sympathetic concern*. When infants are observed with peers who become distressed, the bystander infants gesture to and touch the distressed peer and turn their gaze toward the peer's mother, not their own mothers (Hay et al., 1981; Liddle et al., 2015). When 8‐ to 10‐month‐olds observed distressed peers, their facial expressions and gestures signified concern for the upset peers, not just contagious crying (Roth‐Hanania et al., 2011). One‐ and two‐year‐old children actively try to comfort people who are distressed (Demetriou & Hay, 2004; Lamb & Zakhireh, 1997; Zahn‐Waxler et al., 1992). These empirical findings do not in themselves provide proof for an evolutionary perspective, but they are compatible with de Waal's (2008) theory of empathy.

#### Theories of Helping

An evolutionary perspective has also been brought to bear on the evidence that very young children help other people obtain their goals, a phenomenon that has been reported in “baby biographies” (e.g., Church, 1966) and a classic observational study in a laboratory setting designed to look like a home (Rheingold, 1982). In experiments conducted by Warneken and Tomasello (2006), 18‐month‐olds observed adults drop objects that rolled out of reach or try to overcome physical obstacles to complete a task. Most of the 18‐month‐olds helped with at least one task, without any praise or other reward. Such spontaneous helping is observed in infants as young as 14 months (Warneken & Tomasello, 2009). Laboratory chimpanzees who took part in analogous tasks helped experimenters in some but not all of the tasks.

Children's spontaneous helping seems intrinsically motivated (Rheingold, 1982). Positive effects of modeling on 16‐month‐olds’ helping is task‐specific and influenced by prior interactions with the person who is in need of help (Schuhmacher et al., 2019). Helping is not dependent on social reinforcement. Indeed, attempts to reward toddlers for helping actually undermines their helpfulness (Warneken & Tomasello, 2008).

However, current theorists argue for an interactionist perspective on helping that acknowledges children's social experiences (e.g., Dahl, 2019). The pleasure that 1‐year‐olds take in helping others draws upon positive experiences they have with their caregivers. Patient parents often encourage their children and scaffold their attempts to help, despite the fact that the delays caused are objectively unhelpful (Dahl & Brownell, 2019).

#### Theories of Sharing

In addition to demonstrating empathy and helping, most infants share what they see and find by pointing out, showing, and offering objects to other people (Rheingold, 1973). Social learning theory provided the framework for studies of older children's sharing (e.g., Bryan & London, 1970). However, as was the case for helping, social reinforcement and modeling does not significantly influence infants’ sharing (Hay & Murray, 1982; Rheingold, 1973) and training infants to point does not increase pointing (Matthews et al., 2012). Rather, infants’ sharing emerges in tandem with other skills that contribute to language and communication (e.g., Bates et al., 1975; Tomasello et al., 2007). Thus, along with empathy and helping, sharing qualifies as a developmental milestone that emerges by the first birthday and is not easily explained by social learning theory. However, becoming aware of when and where and what to share certainly depends on children's experiences in their families and cultures.

### Research Topics

#### Separate Dimensions of Prosocial Behavior

Different forms of prosocial behavior do not necessarily correlate with each other (Eisenberg & Fabes, 1998; Radke‐Yarrow et al., 1983). Empathy, helping, sharing, and comforting emerge at different ages and appear to be supported by different sets of cognitive and social skills. Underlying motives may differ. Paulus (2014) proposed that different motivations underlie three different types of prosocial behavior (helping, sharing, and comforting) and that different types of cognitive representations support each type. For example, comforting someone would require empathic motivation, the ability to notice and comprehend another person's emotional signals, and ultimately moral understanding.

Similarly, Dunfield and Kuhlmeier (2013) noted that different types of prosocial behavior require young children to pay attention to different sets of cues, such as emotional distress (which might provoke empathic concern), instrumental need (which might evoke helping), and desire for particular resources (which might evoke sharing). The different types of prosocial behavior are not equally likely to occur and do not necessarily correlate with each other. For example, in a set of studies, 2‐ to 4‐year‐old children were more likely to respond helpfully to an experimenter's need for help than to other opportunities for prosocial action (Warneken & Tomasello, 2009). Even by 4 years of age, not all children were responsive to people's desires for resources or their need for comfort. Indeed, in later research, Paulus et al. (2013) found that 18‐month‐olds’ instrumental helping was negatively associated with comforting.

#### Other Biological Influences

Even within the evolutionary perspective on prosocial behavior, more proximal biological processes underlie individual differences. Young children's prosocial behavior is influenced by their genetic heritage as well as their environments. Findings from a large, nationally representative twin study identified a genetic contribution to 2‐year‐olds’ prosocial behavior, as reported by parents (Knafo & Plomin, 2006). In that sample, the extent of genetic influence increased as the children grew older while the influence of the shared family environment declined.

More recent work suggests that genetic factors contribute to children's empathic concern and comforting, but not their sharing, cooperation, and helping. Infant temperament, which is known to be heritable (Goldsmith, 1996), is associated with comforting, but not instrumental helping (Schuhmacher et al., 2017). Temperamental fearfulness is negatively correlated with empathic concern (Van der Mark et al., 2002).

Children's risk for neurodevelopmental disorders may also influence their perception of other people's emotions. For example, in a study that contrasted toddlers who were at high and low genetic risk for autism (i.e., those who did or did not have a sibling who had been diagnosed with autism), the lower‐risk toddlers showed more empathic concern in response to a crying baby and to an adult pretending to be in pain. This finding was not completely explained by group differences in language and communication skills (Campbell et al., 2015).

Another biological mechanism thought to be related to empathy is the hormone oxytocin (e.g., Uzefovsky et al., 2015). For example, in an experimental study, adult women who had been given oxytocin were more likely to respond to infants’ crying (Riem et al., 2011). Molecular genetic analyses revealed a link between the oxytocin receptor gene and preschool‐aged children's helping and comforting but not sharing (Wu & Su, 2014). In general, the evidence suggests that a set of genetic factors related to emotion regulation and empathy may contribute to individual differences in feeling for and ministering to others, but not sharing resources or helping with tasks.

Neuroimaging findings similarly indicate that different mechanisms underlie helping versus comforting. In a longitudinal study of 34 infants (Paulus et al., 2013), resting state brain activation was measured by electroencephalogram (EEG) at 14 months of age. At 18 months, the infants took part in helping tasks. At 24 months, their mothers simulated pain. Brain activation patterns predicted both helping and empathic concern for their mothers’ pain, but in different ways. Helping was associated with right brain activation in the temporal lobe, whereas empathic responses to the mother's pain was associated with left brain activation in the frontal lobe. EEG patterns were also associated with the infant's age, sample retention, and task characteristics, suggesting that different mechanisms support different types of early prosocial behavior.

#### Social Experiences

Social influences on early prosocial behavior extend beyond modeling and social reinforcement. Emotion socialization is important. For example, parents’ talk about emotions with their 1‐ and 2‐year‐olds fosters prosocial behavior (Brownell et al., 2013).

In adult life, prosocial behavior is selective, affected by many contextual and social psychological factors (e.g., Darley & Latané, 1968). Similarly, prior experience with particular people influences early prosocial behavior (Demetriou & Hay, 2004; Kuhlmeier et al., 2014). Children gradually come to understand that it is good to share, cooperate with, and help others, but it is not always socially appropriate to do so. Cultural differences influence the nature and extent to which parents engage in the socialization of prosocial behavior (e.g., Kärtner et al., 2010). For example, culture affects the way in which parents scaffold their toddlers’ attempts at helping (Köster et al., 2016).

#### Gender

Biological sex and gendered social experiences might both have an impact on children's prosocial behavior. Although there are strong theoretical claims about the role of gender in adults’ prosocial behavior (e.g., Eagly, 2009), the evidence for gender differences in early life is mixed. In some studies, gender differences are found for some age groups and some tasks but not others (e.g., Warneken & Tomasello, 2013). In still other studies, there is no evidence for gender differences (e.g., Ensor et al., 2011), and in others, associations with gender are bound up in more complex interactions (e.g., Dunfield & Kuhlmeier, 2013).

Different sources of information produce contradictory findings. Gender differences are detected in parents’ reports but not in direct observation (e.g., Gross et al., 2015). It is possible that many observational studies do not have sufficient power to test for gender differences or interactions between gender and other variables. It is also possible that parents’ judgments are influenced by gender stereotypes.

#### Continuity and Change Over Time

As was the case for aggression, children's prosocial behavior reveals patterns of continuity as well as change over time. Developmental trajectories for helping and comforting were identified in a large, nationally representative Canadian sample (Nantel‐Vivier et al., 2014) where children's behavior had been reported on between 2 and 11 years of age. The analyses identified three trajectory groups representing low, medium, and high levels of prosocial behavior across time. Continuity and change in prosocial behavior over time were also seen in a large, nationally representative study of British twins (Knafo & Plomin, 2006). Genetic factors contributed to continuity from 2 years of age to later childhood, whereas environmental factors led to change.

## The Association Between Aggression and Prosocial Behavior

Aggression and prosocial behavior could be associated in different ways: as opposite ends of a single dimension; as two separate dimensions that are negatively correlated; as separate dimensions that are not significantly correlated; or as two forms of social behavior that are quite distinct from each other but nonetheless positively correlated. Different patterns may be found under different conditions and at different points in the life span. The statistical and theoretical significance of any associations that are found will depend on the definitions that are used, the context of the study, and other features of the research designs in which prosocial behavior and aggression are studied.

In the first wave of studies of nursery school children's social behavior in the early 20th century, some investigators found that prosocial behavior was positively correlated with aggression. For example, in a study of quarrels and friendship patterns in a nursery school, Green (1933) found a moderate, positive association between quarreling and friendly behaviors, *ρ* = .34, and concluded that “Quarreling is a part of friendship rather than its antithesis” (p. 248). Similarly, in a study of nursery school children, Murphy (1937) discovered that aggressive behavior was positively associated with the display of sympathy.

Some decades later, at the beginning of a substantial program of work on prosocial development, Yarrow noted that the association between young children's prosocial behavior and their aggression was complicated and influenced by gender and individual differences (Yarrow et al., 1975). In a study based on detailed home observations, a positive association between prosocial behavior and aggression was found for girls, regardless of how much aggression they engaged in. However, for boys, that positive association was seen only in those whose level of aggression was below the mean for their gender. Yarrow and colleagues concluded that boys’ use of force at low levels would more accurately be classified as assertive rather than as hostile, and thus reflected general sociability.

As already noted, recent studies have tended to focus on either prosocial behavior or aggression, rather than making direct comparisons of the rates of the two kinds of behaviors, or examining patterns of association between them. Even when prosocial behavior and aggression are measured in the same samples, the findings tend to be published separately. This publication strategy means that the associations between the two types of behavior are rarely scrutinized.

When prosocial behavior and aggression are studied simultaneously, a negative association between prosocial and aggressive behavior is evident in middle childhood (e.g., Romano et al., 2005; Strayer & Roberts, 2004), although that finding may also reflect differences in the way in which the two constructs are assessed (Hay & Pawlby, 2003). In contrast, in younger children, prosocial behavior may be positively associated with aggression (Garner & Dunsmore, 2011; Gill & Calkins, 2003), or the correlation may not be significant (Persson, 2005).

Recently, prosocial behavior and aggression were studied together by investigators of a large Canadian sample (Nantel‐Vivier et al., 2014). The researchers analyzed joint trajectories of both behaviors longitudinally as children progressed from 2 to 11 years of age. The most common joint trajectory, in 22% of the sample, showed a moderate level of aggression coupled with a moderate level of prosocial behavior. However, 46% of the children on the high‐aggression trajectory showed very low levels of prosocial behavior. These patterns suggest that aggression and prosocial behavior are negatively associated at the extremes but not in the middle of the distributions.

Learning more about the association between prosocial behavior and aggression has practical implications because the need to promote the former and reduce the latter places demands on parents, childcare providers, and teachers. For example, in a short‐term longitudinal study of caregivers’ adult‐centered and child‐centered approaches to 1‐year‐olds’ peer interactions, the caregivers used both adult‐centered and child‐centered scaffolding strategies (Williams et al., 2010). Adult‐centered strategies included distraction, such as moving a toy that two children wished to play with or moving one toddler physically away from other peers. Child‐centered strategies included talking about peers and attempts to include peers in activities. Adult‐ and child‐centered strategies were positively associated, but adult‐centered distraction techniques predicted lower levels of sociability with peers later on. Suppression of conflict might have had the unintended effect of suppressing toddlers’ attempts to socialize with their peers.

In sum, previous studies provided only limited evidence for associations between prosocial behavior and aggression. These behaviors have been studied in different theoretical traditions, using different sampling strategies and research methods. To extend our knowledge about the association between prosocial behavior and aggression, we now report longitudinal findings about their parallel development from infancy to 7 years of age. Because of the importance of the family environment that parents create, we began the study when mothers were pregnant with their firstborn children.

## II General Methods

## Design

Our analyses of prosocial behavior and aggression draw upon data from the Cardiff Child Development Study (CCDS), a prospective longitudinal study of a volunteer sample of parents and their firstborn children. The study design is a version of a multitrait/multimethod study (Campbell & Fiske, 1959) in which two types of behavior are studied in parallel, using multiple sources of information to measure each behavior. The design yielded information about the parallel development of prosocial behavior and aggression.

**Figure 1:**
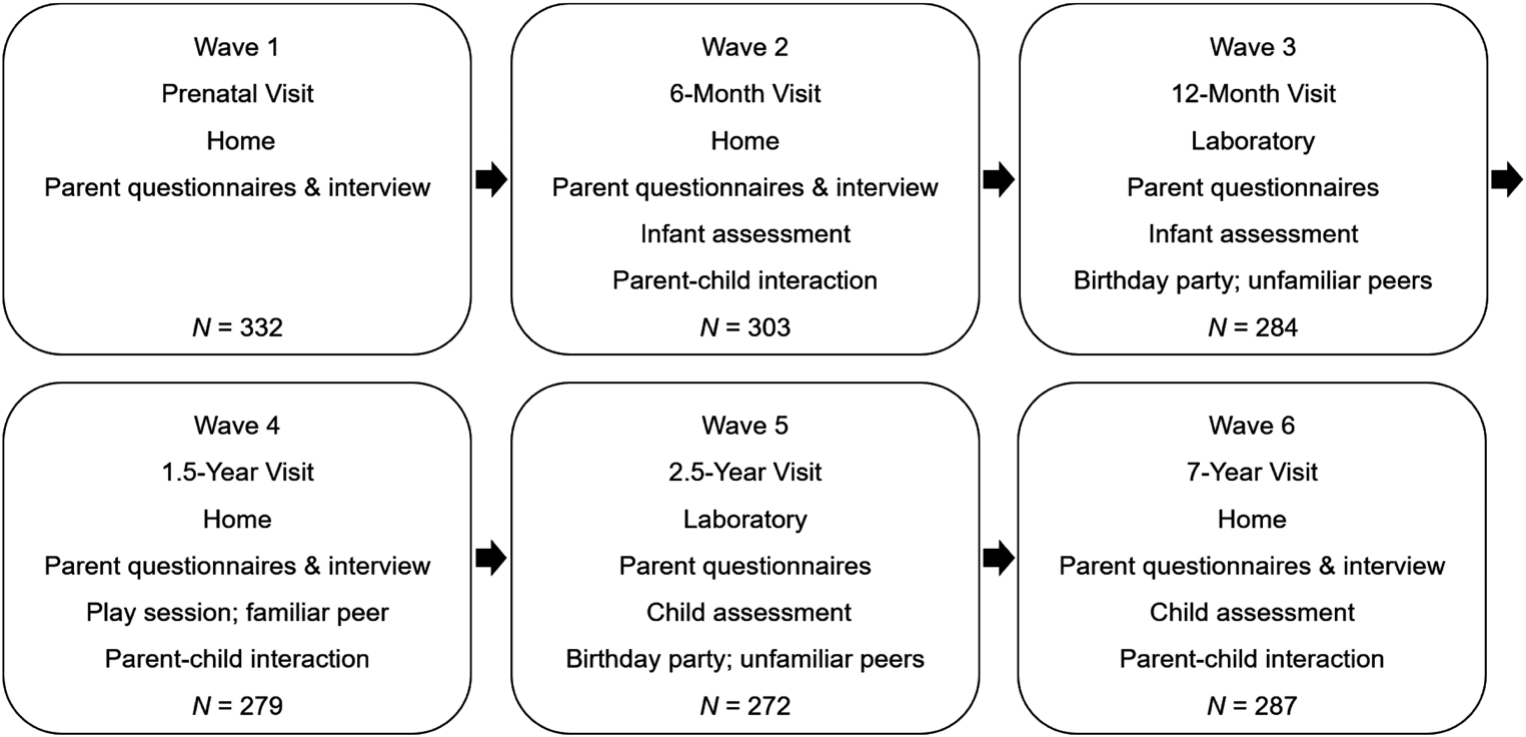
Design and summary of procedures of the Cardiff Child Development Study.

First‐time mothers were recruited during the third trimester of pregnancy (Wave 1). After infants were born, an alternating sequence of home and laboratory visits were scheduled during nonoverlapping target age ranges: 5–7 months (Wave 2), 11–13 months (Wave 3), 18–24 months (Wave 4), 30–36 months (Wave 5), and 6.5–7.5 years (Wave 6). Figure 1 provides an overview of the design and procedures at each wave. Ethical approval was obtained from the National Health Service (NHS) Multi‐Centre Research Ethics Committee and the Cardiff University School of Psychology Research Ethics Committee.

## The Sample

### Recruitment

A recruitment video (DVD) explaining the study procedures was shown to the NHS midwifery teams who helped recruit the sample. Potential families were given recruitment brochures explaining that we were interested in the child's early development and interactions with family members and peers, with the aim of studying how the child would eventually settle into school.

First‐time mothers (*N* = 332) were recruited between November 1, 2005 and July 31, 2007 from NHS prenatal clinics in hospitals and general practice clinics in Cardiff and Vale University Health Board and the Gwent Healthcare Trust, UK. Midwifery teams also granted access to specialist clinics for pregnancy‐related medical problems and outreach services for vulnerably housed pregnant women, which enhanced the representativeness of the sample.

After consultation with midwives, researchers attended clinic and hospital sessions for expectant mothers, drawing upon the guidance of receptionists who indicated which women were first‐time mothers and therefore suitable for the study. The researchers briefly explained the study, told the women what participation would involve, and then, if they were interested, showed them the recruitment DVD. Each potential participant was given an explanatory leaflet to take home and their contact details were recorded. A postcode analysis of home addresses showed that the potential participants represented the full range of UK sociodemographic classifications of neighborhoods (www.acorn.caci.uk).

Within 2 weeks of the initial contact, the CCDS project administrator phoned the families to provide further information. During the telephone call, the administrator explained to the mothers that, if they were in a couple relationship, their partners were also invited to participate. Of the 332 mothers recruited, 300 (90%) lived with a partner, and in 285 (95%) of those couples, the partners participated (99% being biological fathers and 1% same‐sex partners). Two additional biological fathers who were not in a couple relationship with the mother also participated; thus in 86% of the families, both parents took part in the study.

Families booked their first appointment during the third trimester of the pregnancy (Wave 1). No exclusion criteria were set, either for the pregnancy visit or after the baby was born, except in the case of miscarriage, the infant's death, or the infant's experience of severe health problems that prevented participation in the study. No families were excluded on those grounds.

Of the 332 families who joined the study during the pregnancy, 321 (97%) participated in the study after the child's birth: 301 families (94%) at the 6‐month visit (*M* = 6.6 months, *SD =* 0.9); 291 (91%) at the 1‐year visit (*M* = 12.8 months, *SD* = 1.1); 279 families (87%) at the 1.5‐year visit (*M* = 20.6 months, *SD* = 2.3); 272 families (85%) at the 2.5‐ to 3‐year visit (*M* = 33.6 months, *SD* = 2.5 for laboratory visit and *M* = 36.0 months, *SD* = 9.6 for questionnaires), and 287 families (89%) at the 7‐year visit (*M* = 85.2 months, *SD* = 4.8).

### Representativeness of the Sample

Although the CCDS was a volunteer sample, later analyses showed it to be representative of the United Kingdom as a whole, as shown by analyses that compared the family demographic characteristics of the CCDS sample with the subsample of firstborn children in the large Millennium Cohort Study, the most recent national birth cohort study in the United Kingdom, which had similarly recruited families in the early years of the 21st century (K. Kiernan, personal communication, 2009). Demographic characteristics of the families recruited to the CCDS and interviewed in pregnancy (*N* = 332) are displayed in Table 1.

**Table 1:** Sociodemographic Characteristics of the Cardiff Child Development Study (CCDS) Sample at Entry Into the Study (*N* = 332)

Characteristic	Data at Study Entry
Parent's age in years at participant's birth, *M* (*SD*), range	
Mother	28.15 (6.35), 16.09–42.99
Father	30.81 (6.82), 15.62–56.57
Social class	
Middle class	50.9%
Working class	49.1%
Mother's education	
No qualifications	5.1%
Less than basic secondary education	16.6%
Completed secondary education (age 16)	13.9%
Tertiary qualifications (e.g., A levels)	11.7%
Undergraduate degree	28.0%
Postgraduate degree	24.7%
Cultural identity	
Welsh, English, Scottish, Irish	92.7%
Other	7.3%
Relationship status at child's birth	
Married	50.3%
Cohabiting	33.7%
In a relationship, not cohabiting	6.3%
Single	9.6%
Participating child's gender	
Boy	56.8%
Girl	43.2%

*Note*. % indicates percent of sample.

#### Sociodemographic Variables

General demographic characteristics of the parents (their dates of birth, occupational status, cultural identity, educational attainment, and marital status) were collected during interviews and questionnaires with all mothers and the participating fathers, during the prenatal assessment. The age of the parents at the time of the child's birth was recorded. The *occupational status* of both parents was measured using the Standard Occupational Classification 2000 (SOC2000; Elias et al., 1999), based on the highest ranked employment that the parent ever had at entry into the study; SOC is the standard method for determining social class in the United Kingdom. A dichotomous variable was then created by using the parents’ highest rank of employment on the SOC2000 six‐category scale to characterize them as working class or middle class, in line with SOC2000 definitions.

Parents also reported their self‐defined *cultural identity*, using a standard set of categories recommended for use in Wales at the time; 92.7% of mothers described themselves as Welsh, Scottish, English, or Irish; 3.5% as other European nationalities; 1.6% as Bangladeshi, Indian, or Pakistani; and 2.2% as other identities. The fathers’ self‐descriptions were similar, with 92.7% defining themselves as Welsh, English, Scottish, or Irish; 2.3% as other European nationalities; 0.8% as Bangladeshi, Indian, or Pakistani; 0.8% as African or Afro‐Caribbean; and 3.1% as other identities.

We note that the recommended measure did not lead people of color away from defining themselves as Welsh, English, Scottish, or Irish, so it is not appropriate to use the term “White British.” However, we also note that in the Millennium Cohort Study (MCS), the UK‐wide cohort study that began 5 years before the CCDS, 89% of the parents in the whole sample defined themselves as White; for the Wales subsample in the MCS, 97% did so. These comparisons suggest that the CCDS was likely slightly more diverse than the Welsh population as a whole.

Parents provided information about *educational attainment*, which was dichotomized to indicate whether they had or had not achieved the minimum level of qualifications required for the completion of secondary education at age 16 in the United Kingdom (five General Certificate of Secondary Education examinations grade A*–C or equivalent). The parents also reported on their *relationship status*, that is, whether the mothers were in a stable partnership with the baby's biological father and, if so, whether they were legally married.

Because it was important to have a standardized measurement of family characteristics at the point of transition to parenthood, and not all of the women who had been recruited were in a couple relationship, we constructed a sociodemographic risk index that was based solely on mothers’ reports. The characteristics that contributed to the risk index were: (1) being 19 years of age or under at the time of the child's birth; (2) mother's occupation being classified as working class on the SOC2000 measure; (3) having no secondary school qualifications or fewer than five passing grades on the GCSE examinations or their equivalents; (4) not being legally married; and (5) not being in a stable couple relationship.

A principal components analysis based on the polychoric correlation matrix confirmed that all these categorical variables contributed to a single component, with eigenvalues of 3.84 and 0.68 for the first and second component extracted, respectively. The first component explained approximately 77% of the shared variance. Summary scores derived from this principal components analysis are used as the measure of the family's sociodemographic context in the chapters that follow.

Although the sociodemographic risk score was based on mothers’ reports, it is important to note that in families in which both parents provided information, mothers’ and fathers’ reports were generally highly correlated. Fathers were significantly older than mothers, respectively, *M* = 30.7 years, *SD* = 7.2 vs. *M* = 28.4, *SD* = 6.2, *t*(313) = 8.49, *p* < .001, but their ages were highly correlated (*r* = .74, *p* < .001). If the mother was 19 years of age or under when her child was born, the father was likely to be so as well, *κ* = 0.50, *p* < .001. Mothers and fathers agreed in their reporting of the family's income, *r* = .91, *p* < .001. They did not differ significantly in their education, with the mean age of completing education being 19 years for both parents and the number of GCSE passing grades not significantly different. Indeed, the ages at which mothers and fathers completed their education were significantly correlated, *r* = .50, *p* < .001. The sociodemographic risk index was significantly and negatively related to fathers’ reports of family income (*r* = −.64, *p* < .001), the age at which fathers completed their education (*r* = −.44, *p* < .001), and fathers’ own age at the time of the child's birth (*r* = −.60, *p* < .001).

There were no significant differences in the overall demographics of families originally recruited in the study and those that participated at each subsequent wave (all *p*s > .05). However, we shall note instances in which there was a difference between the sociodemographic profile of the full sample versus the subsample of participants who contributed data on a particular measure at a particular wave of data collection.

## Procedure

The assessments included interviews, questionnaires, experimental tasks, and observational methods, in an alternating sequence of home visits and visits to the Social Development Laboratory at Cardiff University (see Figure 1).

### Wave 1: Prenatal Home Visit (Third Trimester)

Pregnant women and their partners were interviewed in their homes by researchers during the third trimester of the pregnancy. Where possible, the mothers and fathers were interviewed at the same time in different rooms. The interviews included a psychiatric assessment of symptoms of psychopathology using the Schedules for Clinical Assessment in Neuropsychiatry (SCAN; Wing et al., 1990). Also included were assessments of employment history, social support networks, and family history of mental health problems. Wales is a bilingual country within the United Kingdom, and Welsh‐speaking as well as English‐speaking families were recruited. If families expressed a preference to be interviewed in Welsh, a Welsh‐speaking research assistant conducted the interview. Translators or interpreters were employed for some families whose native language was not English or Welsh or for participants who had impaired hearing.

Following the interview, questionnaires that measured lifestyle, general health, relationship quality, fertility history, and behavioral history were given to both parents to complete and return to the university at their convenience. A remuneration of a £20 gift voucher was given to the family upon completion of the visit.

### Wave 2: 6‐Month Home Visit

Parents and infants were visited in the home. During the 2‐hr visit, mothers were interviewed about their experience of labor, obstetric complications, mental health (again using the SCAN), any recent changes in general lifestyle arrangements, and their social network. The infant was filmed during a 25‐min assessment which included three parent–child interaction tasks. The infant wore an Actigraph to monitor physical activity and heart rate (for more details, see Meeuwsen et al., 2019) and a saliva sample was taken. Mothers, fathers and, where possible, a close family member or friend completed questionnaires about the infant's development. Parents’ questionnaires included questions about the parents’ health, lifestyle, life events, and relationships. A £20 gift voucher was given to the family.

### Wave 3: 1‐Year Lab Visit

To provide a context that enabled the infants to interact with peers, three families were invited to the School of Psychology at the same time for a simulated birthday party, along with additional assessments. Those who were unable to do so were asked to complete questionnaires. Infants were first assessed in separate testing rooms while their accompanying caregivers completed questionnaires. The three families were then brought together for the birthday party, which took place in a family room decorated to resemble a living room. As part of an emotion regulation challenge, two costumed characters were present at the party. Infants wore Actigraphs to measure physical activity and heart rate and their saliva was sampled for cortisol at entry to the laboratory, after the individual testing, and at the end of the party (for more details on these measures see Hay et al., 2016). Peer interaction was observed (for more details, see Chapter V). At the end of the party, the children were invited to play a Lucky Dip game, selecting a gift‐wrapped book from the box of balls, and a £20 gift voucher was given to each family.

### Wave 4: 1.5‐Year Home Visit

Two research assistants visited families at home around the midpoint of the second year. First, mothers were interviewed and then asked to cooperate to complete a jigsaw puzzle with their toddlers, which was video recorded. For the second part of the visit, each mother was asked to invite a friend with a child of similar age to their home so that peer interaction could be observed (for details, see Chapter IV). Mothers, fathers, and a third person in the child's life completed questionnaires. At the end of the home visit, families were given a £20 voucher and drawing materials for the child.

### Wave 5: 2.5‐Year Lab Visit

As the children approached their third birthdays, families attended another simulated birthday party. The protocol for the visit replicated the procedure used at Wave 3 but with different (age‐appropriate) toys (for more details, see Chapter V). The children once again wore Actigraphs and their saliva was sampled at entry to the laboratory, after the individual testing, and at the end of the party. The party again ended with a Lucky Dip in which children would search in a bag of plastic balls that contained wrapped presents. Mothers, fathers, and third informants were given questionnaires. At the end of the lab visit, the parent was given a £20 voucher.

### Wave 6: 7‐Year Home Visit

Researchers visited the families in the homes for two 2‐hr sessions on separate days. During both sessions, a research assistant interviewed the primary caregiver (98% mothers) about the child's mental health, the parent's own mental health, and other aspects of the family's lifestyle and social network. While the parent was being interviewed, a second research assistant asked the child to complete various tasks. When required, a third research assistant took part in the home visit, to keep any younger siblings occupied and prevent interference with the child testing and parent–child interaction tasks. At the end of each session, the child and caregiver would take part in family games; in some games, siblings were encouraged to join in.

The parent completed a questionnaire on an iPad during the visit and was asked to give permission for a questionnaire to be sent to the child's classroom teacher. Parents’ questionnaires included questions regarding health, lifestyle, life events, relationships, family structure, and their child's behavior. At the end of the second home visit, a gift voucher of £20 was given to the caregiver along with a book voucher of £10 for the child at the end of the session.

## Measurement of Prosocial Behavior and Aggression

Observational methods, questionnaires, and experimental tasks were used to measure parents’ and children's aggression and prosocial behavior at different points in the study. Information about the measures, including information about reliability and validity, is provided in subsequent chapters.

### Parents’ History of Prosocial and Aggressive Behavior

#### Antisocial Behavior

At the Pregnancy Visit, mothers and fathers completed two questionnaires, one entitled “What I Am Like,” which included a section about their present behavior and personality traits, and a second one entitled “What I Was Like as a Child,” which focused on their past behavior. The “What I Am Like” questionnaire included items from the screening questionnaire for the International Personality Disorder Examination (IPDE; Loranger et al., 1994). This screening questionnaire has been used in the United Kingdom and in community samples, including a large national sample in Australia (Lewin et al., 2005). For the present analyses, a subset of IPDE screening items that corresponded to the DSM‐IV criteria for Antisocial Personality Disorder (ASPD) was identified. Because a diagnosis of ASPD requires a history of juvenile CD, an additional set of items measuring DSM‐IV symptoms of CD was incorporated into the “What I Was Like as a Child” questionnaire. A composite variable was created by summing responses to the IPDE screening items (*deceitfulness*, *impulsivity*, *irritability*, *aggressiveness*, *physical fights*, *arrests*, *recklessness*, *lack of remorse*, and *failure to sustain consistent work*) and items added to the “What I Was Like as a Child” questionnaire (*stealing*, *deceitfulness*, *destruction of another's property*, *truancy*, *defiance*, *anger*, and *physical fighting*). The composite showed acceptable levels of internal consistency for both mothers, *α* = .78, and fathers, *α* = .79.

Although concerns have been raised about the accuracy of retrospective recall, with well‐adjusted participants sometimes underreporting their past problems (Rutter, 1998), the retrospective items were significantly associated with the contemporary items for 320 mothers (*r* = .59, *p* < .001) and 261 fathers (*r* = .56, *p* < .001). Mothers’ and fathers’ reports of antisocial behavior were significantly and positively associated, *r* = .33, *p* < .001. Parents’ reports of their antisocial behavior were further validated by their history of arrest. Mothers and fathers agreed in their reports of mothers’ history of arrest, *κ* = 0.73, *p* < .001, and fathers’ history of arrest, *κ* = 0.75, *p* < .001. Parents’ reports of their antisocial behavior were significantly associated with their history of arrest (for 321 mothers: *r* = .56, *p* < .001; for 261 fathers: *r* = .52, *p* < .001).

#### Prosocial Behavior

During the pregnancy, mothers and participating fathers also reported on their past and present prosocial behavior on the “What I am Like” and “What I Was Like When I Was Young” questionnaires. The following items measured the parents’ current prosocial behavior: *get upset when other people are upset*; *help people*; *give practical help*; *help care for children and pets*; *give up a seat on public transport*; *being a blood donor*; and *donating to charity*. The prosocial items embedded in the “What I Was Like as a Child” questionnaire were *being considerate*; *helping people in distress*; *sharing*; *being helpful with tasks*; *being kind*. Across the two scales, there were 12 prosocial items, rated on an ordinal dimension with three levels.

These 12 items were considered indicators of an underlying dimension. Measurement models were constructed separately for mothers and fathers. We estimated models using the weighted least square estimator using a diagonal weight matrix (WLSMV). We also used a Theta parameterization to estimate residual variances of items and associations between these residual variances. Modification indices were used to explore changes to the measurement model that would improve model fit. For both mothers and fathers, items loadings were estimated freely, while the average factor score and factor variance were restricted to 0 and 1, respectively. Analyses and factor scores were estimated using Mplus 7 (Muthén & Muthén, 1998–2012).

Overall, 320 mothers provided data on their own prosocial behavior. A measurement model with a single underlying dimension for mothers did not initially display good fit. Modification indices revealed some correlations between residual items (e.g., the item *being considerate* and the item *being kind*); modeling of these correlations yielded an acceptable fit to the data. All the factor loadings in this model were positive and significant.

Overall, 263 fathers provided information on their past and present prosocial behavior. The measurement model with a single underlying dimension for fathers did not initially display good fit, due to the fact that the item *I am a blood donor* did not display a significant loading on the underlying dimension. After removal of that item, model fit was acceptable.

As was the case for antisocial behavior, the items from the two scales (“What I Am Like” and “What I Was Like as a Child”) were combined for a composite prosocial behavior score, with Cronbach's *α* = .65 for mothers’ reports and *α* = .68 for fathers’ reports. The factor scores for mothers and fathers were normally distributed. There was a small, nonsignificant correlation between their scores.

### Definitions of Children's Aggressive and Prosocial Behaviors in the CCDS

Although some investigators require an act of force to be intentional before it is coded as aggression (e.g., Parke & Slaby, 1983), others define aggression as an act of physical force without requiring independent information about intent (e.g., Tremblay et al., 2005). Young children's aggression often appears to be fueled by anger, a primary emotion that emerges in the first year of life (Bridges, 1932; Sternberg et al., 1983) with dramatic temper tantrums becoming evident in the toddler years (Potegal, 2000). Therefore, our developmental analyses that begin in infancy focussed primarily on *angry aggressiveness*, rather than on verbal or relational aggression. In addition, Hartup's (1974) distinction between *hostile* and *instrumental* aggression is relevant to the study of young children's conflicts; it is helpful to distinguish forceful attempts to gain the possession of toys versus hitting or kicking other children for other reasons.

The measures of prosocial behavior were informed by our previous review of the literature on prosocial behavior (Hay & Cook, 2007), in which we distinguished three general domains of prosocial responses: (1) *feeling for others* (e.g., showing affection and early empathy), (2) *working with others* (e.g., sharing resources and helping other people complete tasks); and (3) *ministering to others’ needs* (e.g., caregiving and comforting people who are in distress. We used questionnaires completed by multiple informants, observations of peer interaction, social decision‐making tasks, and a clinical interview to measure prosocial behavior and aggression. Details on the operational definitions used at different age points are presented in the following five chapters. Details on other measures that are tested as correlates of prosocial behavior and aggression are presented in the chapters in which those variables are first analyzed.

## Overview of Data Analyses

Data analyses are reported in the next five chapters. In Chapter III, we present evidence for individual differences in prosocial behavior and aggression. We examined stability over time in both behaviors and the association between them, as measured by the questionnaires given to parents and other informants at the 1.5‐year and the 2.5‐ to 3‐year visits.

In Chapter IV, we report observations of the children's prosocial behavior and use of physical force against familiar peers, as recorded during the 1.5‐year home visit. The analyses tested for gender differences, the relative rates of prosocial behavior versus aggression, and associations between informant‐rated and directly observed behavior.

In Chapter V, we report longitudinal analyses of the children's observed prosocial behavior and aggression with unfamiliar peers at the 1‐ and 2.5‐year laboratory visits. SPSS linear models analyses controlled for the effects of the participants being paired with particular peers. We tested for gender differences, change over time, and associations between prosocial behavior, aggression, and the children's other skills.

Chapter VI presents correlational analyses of parents’ and teachers’ reports of children's prosocial behavior and aggression at the 7‐year home visit. Both behaviors are examined in relation to the children's verbal ability, cognitive skills, emotion regulation, and social decision‐making tasks.

In Chapter VII, we report longitudinal analyses that tested whether the children's angry aggressiveness in the second and third years of life predicts high levels of anger, aggression, CU traits, and DSM diagnoses of behavioral disorders made at the 7‐year visit. Additional analyses test whether earlier prosocial behavior protects children against those clinically meaningful outcomes.

In Chapter VIII, we present a summary and discussion of findings. We note the limitations and implications of the study.

## III Prosocial Behavior and Aggression From 1.5 to 3 Years: Informants’ Reports

We analyzed informants’ reports for evidence of change and continuity in prosocial behavior and aggression between 1.5 and 3 years, a time in development when children acquire language, develop new skills, and learn how to regulate their behavior. It is important to understand how individual differences in prosocial behavior and aggression emerge against the background of such fundamental developmental change. To do so, we analyzed informants’ reports of children's prosocial behavior and aggressiveness, to gain a broad view of change and continuity over time. These analyses produced robust multi‐informant measures of each trait, which are examined in relation to many analyses in the chapters that follow.

Parent questionnaires are often used in longitudinal studies of young children's prosocial behavior (Knafo & Plomin, 2006; Scourfield et al., 2004) and aggression (e.g., Alink et al., 2006; Côté et al., 2006; NICHD Early Child Care Research Network, 2004). However, most infancy studies measure dimensions of temperament, but do not include items that directly measure the use of force against others. To identify the first signs of aggression, we developed an age‐appropriate measure that could be used at 6 months of age by mothers, fathers, and other people who knew the infant well (Hay et al., 2010). To reduce social desirability issues, items measuring anger and aggressiveness were embedded into a larger questionnaire about infants’ developmental milestones. Age‐appropriate items were then added to subsequent versions of the questionnaire as children grew older. Our previous analyses showed that individual differences in combined anger and aggressiveness were already evident by 6 months and stable over time (Hay et al., 2014; Perra et al., in press).

We now compare the data on angry aggressiveness with data for prosocial behavior, using a comparable age‐appropriate questionnaire given to parents during the 1.5 and 2.5 year visits. After tracing and following up on the questionnaires sent to families who could not attend the 2.5‐year birthday party, the mean age at which the questionnaires were returned was 3.0 years (*SD* = 0.8), and we subsequently refer to the questionnaire data at this wave as the 2.5‐ to 3‐year assessment. Along with the items measuring anger and aggression, a broad range of prosocial items were included. The multi‐informant design allowed us to generate measurement models of both constructs from the 1.5‐year and 2.5‐ to 3‐year questionnaire data. In longitudinal research, it is recommended to retain the integrity and representativeness of the original population; therefore we used data imputation where appropriate in the analyses of the informants’ reports (e.g., Spratt et al., 2010).

Previous surveys and observational studies had shown that gender differences in social behavior emerge around the second birthday (Baillargeon et al., 2007; Crockenberg et al., 2008; Hay et al., 2011c). We thus predicted that gender differences in prosocial behavior and aggression might not yet be apparent at 1.5 years but would be evident at 2.5–3 years.

## Method

### Participants

These analyses focused on 277 families who provided questionnaire data at Waves 2, 4, or 5. They did not differ significantly from the overall sample on the sociodemographic risk index.

### Measures

#### CCDS Developmental Milestones Questionnaires (CCDSMSQ)

The CCDSMSQ was a checklist measuring age‐appropriate developmental milestones (e.g., motor and communication skills), which were drawn from the handbook for the Bayley Scales (Bayley, 2006), and prosocial, anger, and aggression items were added. Age‐appropriate versions of the questionnaires were developed for the different waves of assessment. The questionnaires were given to mothers, fathers, and a third family member or friend who knew the child well.

To reduce demand characteristics that might bias informants’ reports, age‐appropriate items measuring prosocial and aggressive behaviors were embedded in the CCDSMSQ at each wave. These items were used to construct two scales to measure angry aggressiveness and prosocial behavior, respectively.

#### Cardiff Infant Contentiousness Scale (CICS; Hay et al., 2010)

The CICS is a 4‐item subscale that measures *angry aggressiveness* and was first included in the milestone questionnaire at Wave 2. We focused on angry aggressiveness, rather than other forms of verbal or social aggression, because angry aggressiveness is already evident earlier in childhood and therefore could be measured at each wave. The 4‐item CICS, which included the items *angry moods, temper tantrums, hits*, and *bites*, had adequate internal consistency (median *α* across informants = .68, which is comparable to that typically found for measures of children's conduct problems, e.g., Goodman, 2001). There was significant agreement between all possible pairs of informants, with substantial agreement between parents (*r* = .51, *p* < .001).

Because responses on the CICS might be interpreted as parents’ negative attributions about their infants rather than a fair report of the child's actual behavior, the CICS was validated by direct observation. At 6 months, the CICS score was significantly associated with the infant's observed distress in response to frustration (being strapped in a car seat, an item adapted from the LabTAB; Clark et al., 2000). The CICS also significantly predicted the infant's observed use of force against peers 6 months later (Hay et al., 2011b).

At the 1.5‐year and 2.5‐ to 3‐year assessments, the four CICS items were again included in an expanded version of the CCDSMSQ suitable for older children. Two new age‐appropriate items that measured instrumental aggression were added: *grabbing toys out of other children's hands* and *hitting/kicking to get toys*. With the inclusion of these items, the intentionality of behavior becomes apparent (see Hay et al., 2011a). The informants’ mean rating was validated by the children's observed tendencies to use physical force against their peers at the 1.5‐year home visit and 2.5‐year laboratory visit (see Chapters IV and V for more details).

Of the 321 families remaining in the sample after the child's birth, 277 families (86%) returned at least one checklist from at least one informant (274 mothers, 219 fathers, and 239 third informants) at each wave of assessment. The 6‐item CICS showed internal consistency within each group of informants at both Wave 4 (mean *α* across the informants = .75, ranging between .72 and .77) and Wave 5 (mean *α* = .74, ranging between .74 and .75). All informants’ reports were significantly associated with each other at each wave. Inter‐rater correlations for the 6‐item CICS ranged from *r* = .48, *p* < .001 for mothers and fathers at the 1.5‐year visit to *r* = .27, *p* < .001 for fathers and third informants at the 2.5‐ to 3‐year assessment.

#### The Cross‐Informant Angry Aggressiveness Scale

To obtain summary angry‐aggressiveness scores that drew on the different informants, we computed a composite score for each of the CICS items by averaging across informants who had completed the CICS at Waves 2, 4, and 5. These scores were then categorized in three brackets (coded 0, 1, and 2), using cut‐point scores of 0.65 and 1.65 to round the average score up to the next category.

To assess changes over time, we developed a longitudinal measurement model of angry aggressiveness. In this model, the composite categorial items at Waves 2, 4, and 5 were considered indicators of underlying angry aggressiveness in the first, second, and third years of life, respectively. We tested different models to investigate measurement invariance. In developing and testing this model, we used the robust WLSMV to take into account the categorical nature of the items. We opted for a Theta parameterization, which allows for the estimation of residual variances of categorical items. The analyses were conducted using Mplus 7 (Muthén & Muthén, 1998–2012). Initial runs of the model indicated problems related to a linear dependency between items *hitting*/*kicking* and *biting* at Wave 5. To account for this dependency and the association between these items, the item *hitting*/*kicking* at Wave 5 was not considered as an informative indicator, and therefore removed from the analyses.

Different measurement models were tested on 304 participants whose informants provided valid milestone data on at least one occasion at Waves 2, 3, 4, or 5 (95% of the families seen after the birth of the child). The final model was a partial‐invariance model: Item loadings were invariant across age, with the exception of the item *temper tantrums* at Wave 2 and the item *biting* at Wave 5. The thresholds of the invariant items (i.e., the points at which a hypothetical underlying continuous distribution is divided into categories with different frequencies) were the same across measurement occasions. A further parameter included in this model was a significant association between the residual variance of items *hitting/kicking* and *grabbing toys* at Wave 4. The mean and variance of the underlying factor at Wave 2 were fixed to allow model identification but were free to vary on other measurement occasions. The results indicated a large increase in factor scores by Wave 4 and a further increase at Wave 5. Overall, this model demonstrated good fit to the data: comparative fit index (CFI) = 0.96; Tucker–Lewis index (TLI) = 0.96; root mean square error of approximation (RMSEA) = 0.04; weighted root mean square residual (WRMR) = 0.96; Model *χ* ^2^(90) = 146.08, *p* < .001. Therefore, this model was used to estimate angry‐aggressiveness factor scores for those participants (*N* = 304) who provided valid data across the study.

#### Cardiff Child Prosocial Scale (CCPS)

Age‐appropriate items designed to measure prosocial behavior were included in the milestone questionnaires given out at the 1.5‐year and 2.5‐ to 3‐year visits (Waves 4 and 5). Although some individual items were repeated across these two waves, new items were introduced at the older age to capture the emergence of different types of prosocial behaviors over time.

At the 1.5‐year visit, the mothers, fathers, and third informants were asked to report on their children's empathy, sharing, and cooperative play. The seven age‐appropriate prosocial items were: *gets upset when another person is upset*; *comforts others*; *shows affection*; *shows objects*; *offers toys to other children*; *plays games*; *plays ball games*. Informants responded by indicating the frequency of the behavior in three categories using the options of “Not yet,” “Sometimes” and “Often.” The reports obtained from the three different informants each indicated a single prosocial dimension, with Cronbach's *α* for mothers’, fathers’, and the third informants’ reports being .65, .62, and .66, respectively. A total prosocial score was calculated for each informant by summing the item scores. To allow for some missing items, the total scores were prorated. There was significant agreement between mothers and fathers (*r* = .47, *p* < .001); between mothers and the third informants (*r* = .30, *p* < .001), and between fathers and third informants (*r* = .28, *p* < .001).

At Wave 5, one prosocial item (*shows objects*) was dropped, two were slightly reworded, and five additional age‐appropriate items were added. Therefore, the 10 prosocial items in this version of the questionnaire were: *gets upset when another person is upset*; *comforts sad people*; *shows affection*; *is considerate*; *offers toys to other children; shares*; *is helpful*; *is kind to other children*; *plays cooperative games*; and *plays ball games*. As had been the case for the 1.5 year‐olds, the prosocial items indicated a single dimension, with Cronbach's *α* for mothers, fathers, and third informants being .76, .79, and .76, respectively. The prosocial ratings showed significant agreement between mothers and fathers (*r* = .47, *p* < .001); between mothers and third informants (*r* = .32, *p* < .001); and between fathers and third informants (*r* = .29, *p* < .001).

#### The Cross‐Informant Prosocial Behavior Scale

Based on these preliminary analyses, a cross‐informant composite score was generated for each of the prosocial items by averaging scores across informants, categorizing these scores in three brackets (coded 0, 1, and 2), using cut points scores of 0.65 and 1.65 to round the average score up to the next category.

Some categories of response had very few cases. For example, all but one 1.5‐year‐old showed objects. Consequently, to avoid problems with model convergence and estimation of parameters, cells with frequencies under 10 were collapsed with the adjoining cell in the matrix. At Wave 5, only nine 2.5‐ to 3‐year‐olds did not show affection often; because of the sparse distribution of participants in this item category and related problems in model convergence, showing affection was excluded from the indicators of prosocial behavior at that age.

The composite items measuring prosocial behavior, averaged across all possible informants for each child, indicated reliability at the 1.5‐year (*α* = .64) and the 2.5‐ to 3‐year assessments (*α* = .78). The composite items were thus considered to be indicators of underlying prosocial dimensions at each age. We tested different models to investigate measurement invariance. Because the indicators were categorical, we considered the robust WLSMV with a Theta parameterization, which allows the estimation of residual variances of categorical items. The analyses were conducted using Mplus 7 (Muthén & Muthén, 1998–2012). Additional details on all the preliminary models tested prior to the final models that are reported below are available from the authors.

We tested different models with increasing constraints on the measurement parameters. Model 1 considered no measurement invariance of the items, so that factor loadings on the underlying dimensions differed across measurement occasions. Model 2 constrained factor loadings of items used across measurement dimensions to be consistent across measurement occasions. In addition, the thresholds of the items (i.e., the point at which a hypothetical underlying continuous distribution is divided into categories with different frequencies) were also invariant across measurement occasions for those items used at both waves. This model was then expanded to allow associations between residual variances of some items (Model 3). The three models were estimated on 288 participants for whom data were available at either wave.

The final revised measurement invariance model (Model 3) provided the best fit to the data and was thus retained: CFI = 0.95; TLI = 0.94; RMSEA = 0.05; WRMR = 1.06; Model *χ* ^2^ = 170.35, *p* < .001. Apart from the measurement invariance constraints, Model 3 allowed an association between the residual variances of the 2.5‐ to 3‐year items *offers toys* and *shares*, *helpful* and *comforts others*, and *helpful* and *gets upset when others are upset*. Furthermore, an association was modeled between residual variances of the item *gets upset when others are upset* at 1.5 years and the item *helpful* at 2.5–3 years.

Overall, the measurement model indicated increasing factor scores in the underlying dimensions at both time points. Factor scores were estimated for the 288 children with data on prosocial behavior that were available at both waves.

## Results

### Continuity in Individual Differences

The informants reported that individual differences in both types of social behavior showed continuity over time. Previous analyses had shown that informants’ ratings of angry aggressiveness at 1.5 years predicted angry aggressiveness at 2.5 years (*r* = .58, *p* < .001; see Hay et al., 2014). Continuity over time was now seen in the children's prosocial behavior as well. Ratings of prosocial behavior on the CCPS at 1.5 years predicted ratings of prosocial behavior at the 2.5‐ to 3‐year assessment (*r* = .68, *p* < .001).

### Associations Between Prosocial Behavior and Aggression

The next set of analyses addressed the association between prosocial behavior and aggression at both ages. Despite the stability in individual differences in both behaviors, the nature of their association did change over time. At the 1.5‐year visit, aggressiveness scores were not significantly correlated with prosocial scores (*r* = .04, *ns*). In contrast, by the time of the 2.5‐ to 3‐year assessment, the two variables were negatively correlated, *r*(288) = −.12, *p* < .05. An *r* to *z* transformation for dependent samples (*N* = 288) indicated that these two correlation coefficients were significantly different (*z* = 1.92, *p* < .05).

### Correlates of Angry Aggressiveness

In earlier work, we identified predictors of the children's CICS scores, notably including the sociodemographic risk index, parents’ own history of antisocial behavior, and mothers’ experience of prenatal depression (Hay et al., 2014; Perra et al., in press), and we thus do not repeat those analyses here. However, to address the current questions, we conducted parallel analyses of the aggressive and prosocial factor scores at the 1.5‐year and 2.5‐ to 3‐year assessments.

#### Prediction of CICS Scores at 1.5 Years

In an initial analysis, the children's aggressive factor score at the 1.5‐year visit was regressed on the family sociodemographic adversity score, the mother's history of antisocial behavior, the child's gender, and the child's precise age in months. Univariate correlations are reported in Table 2. The only significant predictor of angry aggressiveness at the toddlerhood assessment was the mother's history of antisocial behavior, *β* = .29, adjusted *R* ^2^ = .07, *F*(4, 261) = 6.05, *p* < .001. In an additional analysis of the subset of families in which fathers had reported on their own behavior, both parents’ histories of antisocial behavior predicted the child's aggressiveness, *β* = .21 for mothers and *β* = .14 for fathers, adjusted *R* ^2^ = .05, *F*(5, 222) = 3.49, *p* < .01.

**Table 2:** Pearson‐Product and Point Biserial Correlations Between Children's Prosocial Behavior, Gender, Family Risk Factors, and Children's Aggressiveness at the 1.5‐Year Visit

	Child's Prosocial Behavior	Gender	Sociodemographic Risk Index	Mother's Antisocial History	Mother's Prosocial History	Child's CICS Aggression
Child's prosocial behavior		−.15*	−.02	−.01	.19**	.08
Gender			.10†	.09	−.05	−.05
Family adversity				.51***	−.24**	.09
Mother's antisocial history					−.29***	.26***
Mother's prosocial history						−.06
Mean	−0.02	1.57	0	4.37	16.06	0
*SD*	0.79	0.50	1	4.01	3.41	1.85

*Note*. Gender is coded as girls = 1 and boys = 2.

CICS = Cardiff Infant Contentiousness Scale (Hay et al., 2010).

^†^ *p* < .10.

* *p* < .05.

** *p* < .01.

*** *p* < .001.

#### Prediction of CICS Scores at 2.5–3 Years

In the next analysis, the children's CICS scores at the 2.5‐ to 3‐year assessment were regressed on the family's sociodemographic risk score, the child's gender, the child's precise age in months, and the mother's history of antisocial behavior. Univariate correlations between these variables are presented in Table 3. Once again, the only significant predictor of the child's angry aggressiveness was the mother's own history of antisocial behavior, *β* = .19, adjusted *R* ^2^ = .06, *F*(4, 248) = 5.16, *p* < .01.

**Table 3:** Pearson‐Product and Point Biserial Correlations Between Child's Prosocial Behavior, Gender, Family Risk Factors, and Child's Aggressiveness at the 2.5‐ to 3‐Year Visit

	Child's Prosocial Behavior	Gender	Family Adversity	Mother's Antisocial History	Mother's Prosocial History	Child's CICS Aggression
Child's prosocial behavior		−.21***	−.11†	−.04	.24***	−.21**
Gender			.10†	.09	−.05	.12†
Family adversity				.51***	−.24**	.16*
Mother's antisocial history					−.29***	.25***
Mother's prosocial history						−.06
Mean	1.15	1.57	0	4.37	16.06	0
*SD*	0.88	0.50	0.99	4.01	3.41	0.78

*Note*. Gender is coded as girls = 1 and boys = 2.

CICS = Cardiff Infant Contentiousness Scale (Hay et al., 2010).

^†^ *p* < .10.

* *p* < .05.

** *p* < .01.

*** *p* < .001.

In a subsequent analysis of the subsample in which fathers had reported on their own antisocial behavior, both parents’ histories of antisocial behavior predicted their child's angry aggressiveness, *β* = .19 for mothers and *β* = .15 for fathers, adjusted *R* ^2^ = .08, *F*(5, 203) = 4.81, *p* < .001. In sum, the children's CICS scores were not significantly associated with the child's age or gender, or the sociodemographic risk score, but were predicted by their parents’ own antisocial behavior.

### Correlates of Prosocial Behavior

#### Prediction of Prosocial Scores at the 1.5‐Year Visit

In an initial analysis, the children's prosocial score at the toddlerhood assessment was regressed on the family sociodemographic adversity score, the mother's history of antisocial behavior, the mother's history of prosocial behavior, the child's gender (with girls coded as 1 and boys as 2), and the child's age at the time of the 1.5‐year visit (see Chapter II for description of these predictors), with the child's current CICS score entered at the second step of the analysis. Univariate correlations between these variables are presented in Table 2.

The regression analysis based on a sample of 262 families with information about mothers’ own prosocial behavior indicated that the toddlers’ prosocial behavior was significantly predicted by the child's gender, with higher prosocial behavior associated with being a girl (*β* = −.15), with being older (*β* = .19) and with the mother's history of prosocial behavior (*β* = .18), adjusted *R* ^2^ = .08, *F*(4, 257) = 5.22, *p* < .001. The sociodemographic risk score, the mothers’ history of antisocial behavior, and the child's angry aggressiveness score in toddlerhood did not contribute significantly to the model.

The analysis was repeated on the subsample in which 223 fathers’ self‐reports of prosocial and antisocial behavior were available. Once again, at the 1.5‐year assessment, prosocial behavior occurred at higher rates in girls (*β* = −.17), relatively older children (*β* = .15), and children whose mothers were more prosocial (*β* = .22), adjusted *R* ^2^ = .11, *F*(6, 216) = 5.40, *p* < .001.

#### Prediction of Prosocial Scores at the 2.5‐ to 3‐Year Assessment

In two comparable analyses, the children's prosocial scores were regressed on the same predictor variables: the sociodemographic risk index, the mother's history of antisocial behavior, her history of prosocial behavior, the child's age, and the child's gender were entered at the first step, with the child's current aggressiveness score added at the second step. At this wave, the only significant predictors of prosocial behavior were gender, with girls more prosocial (*β* = −.19) and age in months (*β* = .16), adjusted *R* ^2^ = .11, *F*(6, 245) = 6.38, *p* < .001. Univariate correlations between pairs of variables are presented in Table 3.

The analysis was repeated on the subsample in which fathers had provided information about their own prosocial and antisocial behavior. In that subsample, the child's prosocial behavior was positively predicted by gender, with girls more prosocial (*β* = −.20), the mother's history of prosocial behavior (*β* = .26), and the father's own history of both prosocial (*β* = .15) and antisocial behavior (*β* = .18), adjusted *R* ^2^ = .14, *F*(8, 200) = 5.16, *p* < .001.

## Discussion

The foregoing analyses suggested that, over the first 3 years, children's prosocial actions and aggression gradually disentangle from each other, which corroborates earlier findings (e.g., Persson, 2005). In this sample, age and gender were associated with children's prosocial behavior, but not their angry aggressiveness. The broad scope of the informants’ reports has drawn attention to the association of parents’ own behaviors with their children's prosocial behavior and angry aggressiveness, which might reflect both genetic and environmental influence. Paradoxically, children with higher rates of prosocial behavior had fathers with higher levels of both prosocial and aggressive traits. This unexpected finding might imply that socially active fathers have a positive impact on their children's social development, even if the fathers engage in aggression as well as prosocial behavior.

Nevertheless, a degree of caution is required. These findings are based on reports by informants responding to questions about their own and their child's behavior. These data sources have shared methods variance.

Furthermore, parents’ reports about their children may be influenced by their general expectations for children's behavior, which may lead to some self‐fulfilling prophecies when they rate their own children's behavior. In other words, parents’ responses to the questionnaires may reflect social desirability and aspirations for their children, not unbiased ratings of the children's actual behavior.

In this study, we tried to reduce social desirability effects by embedding the prosocial and aggressive items within a list of normative developmental milestones. Furthermore, by giving questionnaires to more than one informant, we hoped to gain a broader view of the child's behavior than would be possible with a single informant. Nonetheless, it is useful to supplement informants’ reports with observational data. In the next chapter, we turn our attention to the children's prosocial behavior and use of force against familiar peers who visit their homes.

## IV Sharing and Aggression at 1.5 Years: Home Observations

The review of the literature in Chapter I showed that the second year is a time when prosocial skills are rapidly developing (see also Brownell et al., 2013), but the use of physical force against others is still part of the 1‐year‐old's repertoire (e.g., Lorber et al., 2018). The informants’ reports examined in the previous chapter provided evidence for stable individual differences in prosocial behavior and angry aggressiveness. We now ask, how do those individual differences manifest themselves in interactions with other children? To address this question, at the 1.5‐year home visit, we replicated a paradigm developed in an earlier study (Hay et al., 2000), in which the mother of a toddler invited a friend and the friend's child to her home.

At the 1.5‐year visit, the informants’ reports were compared with the observational data. Although the informants agreed with each other, it is still possible that their reports were influenced by bias and expectations for the child. Therefore, we asked, were those children who had been rated as highly prosocial be likely to share toys with their guests? Would those children who had been rated as angry and aggressive be likely to use physical force against their guests? Finally, in comparison with the questionnaire data, would the direct observations also show that at 1.5 years, the use of prosocial behavior was unrelated to the use of aggression?

## Method

### Design

The 1.5‐year home visit comprised a brief interview, observation of mother–child interaction, and, if feasible, observation of peer interaction. Mothers, fathers, and third informants completed questionnaires.

### The Sample

#### Focal Children

At the 1.5‐year visit, 279 families participated (88% of those retained after pregnancy); of those, 270 families (97%) were observed at home and completed questionnaires; 9 completed questionnaires and were not observed. Sixteen families (4%) had left the study and 18 families (5%) could not be traced or contacted for this wave. Only 205 were able to recruit a friend with a child to be observed with the focal child. There were no differences on the sociodemographic risk index between those families who could and could not recruit a peer. Ten (5%) of the peer sessions were not included in the analyses because of video problems. Thus, the analyses of peer interactions are based on *N* = 195 families (53% girls). The visiting child is subsequently referred to as the *guest*.

#### Procedure

Parents took part in a brief interview about their current circumstances, followed by an observation of parent–child interaction (working together to complete a jigsaw puzzle), which was followed by unstructured peer interaction. The first twenty minutes of peer interaction were coded.

No restrictions had been placed on the number of other people present, since they would not be excluded from an ordinary visit to the home. For example, in eight families, the focal child's infant sibling was also present.

In 177 families (90%), only one other child and parent participated in the session. However, in 18 cases, more than one child accompanied the visiting parent; the child closest in age to the participant was designated as the guest whose behavior would be coded. Written permission for the guest's inclusion in the filmed observation was provided by the guest's parent. In the case of four twin pairs in the sample, the ‘guest’ was actually the focal child's twin, serving as the familiar playmate.

The families were given two commercially available toys to use, including a plastic kitchen toy and a wooden shape sorter. Parents were told that the children could play with these toys as well as with the focal child's own toys in any way they normally would. The play session ended with a Lucky Dip game in which both children could pick out wrapped presents (packs of felt pens) from a box of balls. A £20 gift voucher was given to each family at the end of the home visit.

### Measures

#### Coding of the Interactions

The peer interactions were video recorded by the researcher using a hand‐held camera, and then coded using the Peer Interaction Coding System (PICS, Hay & Ross, 1982; see Table 4). The PICS had first been developed in an experimental study in which pairs of 21‐month‐olds met in a laboratory playroom (Hay & Ross, 1982); it was further refined for use in home observations (e.g., Hay et al., 2000) and laboratory studies (e.g., Caplan et al., 1991). The creation of a composite data set from studies using the PICS indicated that the coding system was highly reliable between 9 and 36 months (Hay et al., 2011c).

**Table 4:** Example Episodes of Peer Interactions at the 1.5‐Year Home Visit, Coded Using the Peer Interaction Coding System

Participant Child	Guest
	1. **OFFER** red block to participant (but it falls on the floor) and **SP** “ere go [participant name]”
2. **ACCEPT** red block from peer, bends down to floor and picks up the block	
	3. **NR**
4. **REACH** for spatula in peer's hand	
	5. **OFFER** spatula to participant and **SP** “ere go”
6. **ACCEPT** spatula from peer	
1. **APPROACHES** peer	
	2. **SP** unintelligible as she puts a red saltshaker in her bib
3. **OFFER** green saltshaker, putting it into the peer's bib	
	4. **ACCEPT** holds green saltshaker in her bib but red saltshaker drops on the floor
5. **TAKE5** red shaker peer dropped	
	6. **NR**
7. **OFFER** red saltshaker to peer, holding it toward her at the top of her bib again	
	8. **SP** “No, too big” and does not take red saltshaker

*Note*. Take 5, the child picks up an object that the peer has just put down within the last 5 s.

NR = no response; SP = speech.

The PICS captures key features of dyadic and triadic interactions between young peers, which include conflict (Hay et al., 2000; Hay et al., 2011c; Hay & Ross, 1982) as well as sharing and conversation (Hay, 2006; Hay et al., 1991, 1999). Observers identify *episodes* of social interaction between the peers, timing each episode and transcribing the moves, using a standard set of definitions of socially directed acts. To be coded within an episode of interaction, children's socially directed acts need to be accompanied by visual gaze at the recipient and thus distinguished from accidental encounters, such as bumping into peers. Any speech accompanying nonverbal actions was transcribed. The PICS categories include two measures of *physical aggression* (*tugging* on toys and deploying *bodily force* against the peer's body, by hitting, kicking, or pushing) and two measures of *prosocial behavior* (*offering* toys, food, or other objects to the peer and *adding toys* to an array of items the peer is already playing with).

When preparing PICS transcripts, observers noted whether actions were possibly or definitely present, which were given weights of 1 or 2, respectively, to create scaled scores of aggressive and prosocial behaviors. For example, if the observer recorded with high confidence that a child used bodily force against the peer five times, the scale score for bodily force would be 2 × 5 = 10. However, if the observer was not fully confident that one of the child's actions was forceful enough to meet the criteria for bodily force but did not believe it to be a gentle touch, the action would be recorded with the confidence level of 1 and the scale score would be 9. Most actions were weighted “2.”

Independent observers significantly agreed in recording the two aggressive behaviors (*ICC* = 0.92 for tugging on toys, *ICC* = 0.92 for bodily force) and the two prosocial behaviors (*ICC* = 0.96 for offering objects and *ICC* = 0.90 for adding toys). Inspection of the distributions for the four scale scores revealed that they were skewed and kurtotic; a square root transformation was applied, which improved the normality of the distributions and permitted the use of parametric statistics. The prosocial and aggression scores were not significantly associated with the number of peers present during the home observation.

## Results

In this section, we present descriptive information about prosocial and aggressive behaviors and then examine other variables with which they are associated.

### Observed Sharing and Aggression

#### Sharing

Means and standard deviations for the weighted measures of the focal children's sharing and use of force are presented in Table 5. During the observation, 61% offered objects to their guests and 45% added items to arrays of toys the guests were using (i.e., joined in cooperative play with those toys). The two forms of sharing, which were positively correlated (*r* = .30, *p* < .01), were combined for a total score for sharing.

**Table 5:** Means and Standard Deviations of PICS Scores Measuring Prosocial Behavior (Offering Objects and Adding Items to Peer's Array of Toys) and Use of Force (Tugging on Toys and Bodily Force) at the 1.5‐Year Home Visit

	Girls	Boys	Total Sample
Offers objects			
*M*	4.68	3.20	3.87
*SD*	7.14	4.50	5.87
Adds toys			
*M*	2.47	2.36	2.41
*SD*	4.83	4.73	4.76
Tugs on objects			
*M*	3.58	4.93	4.32
*SD*	4.80	6.60	5.88
Bodily force			
*M*	2.30	2.17	2.23
*SD*	5.58	4.58	5.05

*Note*. PICS = Peer Interaction Coding System.

#### Physical Aggression

Means and standard deviations for the weighted measures of the two types of force are also presented in Table 5; 67% of the focal children tugged on toys or other objects and 41% used bodily force. The number of events coded as tugging and bodily force, which were positively associated (*r* = .41, *p* < .001), were combined to create a total score for physical aggression.

### Relative Occurrence of Sharing and Aggression

There was no indication that either aggressive or prosocial behavior were the preferred approach to interaction with the guests. The mean score for total sharing (*M* = 6.28, *SD* = 8.85), did not differ significantly from the mean score of total physical force (*M* = 6.55, *SD* = 8.73).

### Correlates of Sharing and Aggression

#### Gender

Means and standard deviations for sharing and aggression are presented by gender in Table 5. Multivariate analyses that controlled for the guest's behavior revealed no significant gender differences between focal girls and boys in observed prosocial behavior or physical force.

However, the guest's gender was associated with the focal child's use of bodily force; focal children were more likely to strike out at a girl rather than a boy, girl guests: *M* = 1.07, *SD* = 1.38; boy guests: *M* = 0.63, *SD* = 1.01, *t*(180.81) = 2.56, *p* < .05. The guest's gender was not associated with the focal child's rate of sharing or tugging on toys.

#### Sociodemographic Risk Index

Within the subsample that took part in the peer observation, there were no significant associations between the sociodemographic index and the focal children's sharing and aggression. All univariate associations approached zero.

#### Parents’ Aggressive and Prosocial Behavior

Mothers’ and fathers’ reports of their own past and present behavior (see Chapter II) were examined in relation to the children's behavior to peers. Parents’ self‐reports of their own prosocial and aggressive behavior were not significantly associated with the focal child's observed sharing or aggression.

#### Peer Influence

The focal children's peer‐directed actions were influenced by their guests’ behavior. To remove the contribution of genetic resemblance, the four participants who were observed with their twins were not included in this analysis. Intraclass correlation analyses were used to examine evidence for dyadic influence between the focal children and their guests. Both types of prosocial behavior and both types of aggression revealed mutual influence between the peers: *ICC*s = 0.34, *p* < .01 for offers, *ICC*s = 0.50, *p* < .001 for adding toys, *ICC*s = 0.77, *p* < .001 for tugging, and *ICC*s = 0.20, *p* < .05 for bodily force.

### Links Between Direct Observations and Informants’ Reports

#### Identification of Two Dimensions of Peer‐Directed Behavior

Analysis of the informants’ reports at this age had shown that prosocial behavior and aggression were not significantly associated. A similar pattern was seen in the home observations. Although there were no differences in the rates with which sharing and aggression were observed, there was some evidence of a differentiation of these behaviors. A principal components analysis with varimax rotation revealed two orthogonal factors, the use of force and prosocial behavior accounting for 37% and 30% of the variance in focal children's behavior to guests, respectively (see Table 6).

**Table 6:** Component Loadings for Two Orthogonal Factors Derived From a Principal Components Analysis (With Varimax Rotation) of the Peer‐Directed Behaviors at the 1.5‐Year Home Visit

	Factor 1	Factor 2
Peer‐Directed Actions	Use of Force	Prosocial
Offers objects	.105	.792
Adds objects	−.028	.819
Tugs on objects	.828	.083
Bodily force	.845	−.104

Did the interactions with guests reflect the focal children's general prosocial and aggressive tendencies, as reported by the informants? Put the other way round, were the informants’ reports, which might contain some bias, validated by direct observation?

The prosocial scale derived from the informants’ reports (see Chapter III) was significantly and positively associated with the prosocial factor score derived from the PICS (*r* = .18, *p* < .05). In particular, focal children who were rated as prosocial were significantly more likely to add items to arrays of toys that their guests were playing with (*r* = .25, *p* < .001), which may be interpreted as cooperative play with their guests.

In contrast, the informants’ reports on the CICS aggressiveness scale at this age were not significantly associated with the focal children's aggressive factor score on the PICS (*r* = .05, *ns*). Thus, taken together, informants’ reports about prosocial behavior—but not their reports of angry aggressive behavior—were corroborated by the observations.

## Discussion

Most noteworthy in these findings is the failure to find an association—positive or negative—between prosocial and aggressive behaviors, whether the data are informants’ reports or observations with familiar peers. Informants’ ratings were confirmed by direct observation for prosocial behavior but not for aggressiveness, perhaps due to the lower frequency of observed aggression, which attenuated that distribution. Taken together, the observational and questionnaire findings suggest that, in the second year, children engage in both prosocial behavior and aggression with peers.

There was no significant effect of sociodemographic factors, parents’ history of prosocial behavior and aggression, or the focal child's gender on observed prosocial behavior or aggression. Rather, the guest's gender was influential, with girl guests more likely than boy guests to be the recipients of peer aggression. There are likely to be unmeasured variables related to the gender and temperament of the familiar peer that influenced the nature of their mutual interactions.

These findings testify to the potential importance of the interpersonal dynamics of children's early relationships with familiar peers. Young children's sensitivity to individual peers’ behavior may be a harbinger of the peer preferences documented in the sociometric literature (Rubin et al., 2018). In addition, the fact that the hosts were more likely to hit girls than boys implies that gender biases were already developing in the second year, which is a topic for further research.

Children's behavior at home with familiar peers may differ from how they act when meeting unfamiliar children for the first time. In the next chapter, we report findings from longitudinal observations of the experimental birthday parties where focal children meet up with other participants in the study.

## V Change and Continuity From 1 to 2.5 Years: Lab Visits

The previous chapters provided data demonstrating that prosocial behavior and aggression were both evident at the 1.5‐year visit but did not offer evidence that the two were correlated. The informants’ reports indicated that by the 2.5‐ to 3‐year visit, these behaviors had become inversely correlated. To observe the pattern of change in both prosocial behavior and aggression between the first and third birthdays, a short‐term longitudinal study of the children's interactions with unfamiliar peers was embedded into the overall design.

Many studies of peer interaction have focused on play groups or childcare settings (e.g., Green, 1933; Howes & Stewart, 1987; Murphy, 1937; Vandell et al., 1988) but other investigators observed unacquainted infants paired together in laboratory playrooms (e.g., Eckerman et al., 1975; Goldman & Ross, 1978). The laboratory studies simulate a common challenge that young children face when they meet peers for the first time. They might deploy force, show fear, share toys, or simply manifest bewilderment with the whole situation.

Previous studies demonstrated that infants share and cooperate with new peers (e.g., Eckerman et al., 1975; Hay et al., 1991), but also engage in conflict, which sometimes includes aggression (Caplan et al., 1991; Hay & Ross, 1982; Ross & Conant, 1992). Relatively few such studies have recruited representative community samples or conducted longitudinal analyses with the statistical power to identify trends over time.

We designed our work to examine interactions with unfamiliar peers under realistic conditions by holding simulated birthday parties in a laboratory playroom decorated like a family living room. Families in the study sample as well as two other participating families were invited to the laboratory for a simulated birthday party; this paradigm was designed to provide an ethically acceptable yet emotionally challenging situation in which children would meet new peers. Two simulated birthday parties were held, one at the 1‐year lab visit and the second at the 2.5‐year lab visit, thereby creating a short‐term longitudinal study within the overall longitudinal project.

Our aims were to: (1) compare the relative frequencies of sharing and aggression at both waves; (2) chart change and continuity in prosocial behavior and aggression over time; (3) test for associations between prosocial behavior and aggression at each age; (4) examine associations between parents’ prosocial and aggressive traits and the children's behavior with new peers; and (5) test for associations with other developing skills, in particular joint attention at 1 year and self‐regulation at 2.5 years.

The first four aims complement the analyses of time trends and correlates of prosocial behavior and aggression that were reported in Chapters III and IV. The fifth aim examines other domains of development that might affect both behaviors.

During infancy, prosocial behavior emerges in the context of a broader set of communicative abilities. Infants’ sharing is associated with other joint attention skills that contribute to successful communication (Bretherton & Bates, 1979), in particular the ability to point out things to other people (Rheingold et al., 1976). Individual differences in joint attention skills (gaze following, alternating gaze with another person, and pointing) emerge around the first birthday (e.g., Mundy & Gomes, 1998). We predicted that infants with better joint attention skills might be more likely to share with peers.

By 2.5 years, children are better able to communicate but often spend time in situations where they are required to control their behavior. Harmonious interaction with peers is not just a matter of knowing what to do, but also refraining from doing things that might harm or upset another person. Neurobiological accounts of the development of aggression emphasize self‐regulation in response to frustrating situations (Van Goozen et al., 2007). Therefore, at the 2.5‐year lab visit, in the context of an emotionally arousing birthday party, children with better self‐regulation skills might be less likely to use force against their peers.

However, it is not necessarily the case that well‐regulated children will be prosocial. In one longitudinal study, toddlers’ self‐regulation predicted sharing in middle childhood but was not significantly associated with contemporary measures of early prosocial behavior (Paulus et al., 2015). The measurement of both joint attention and self‐regulation in relation to the children's behavior at the parties was designed to extend our understanding of how children respond to new peers at both ages.

## Method

### Design

At Waves 3 and 5 of the CCDS, three families were invited at the same time to the School of Psychology for individual assessments and a simulated birthday party that included an emotion regulation challenge, followed by free play. Although the experimental tasks at each wave were age‐appropriate, the birthday party procedure was identical at both ages.

The target age window for scheduling the 1‐year lab visit was between 11 and 15 months of age (*M* = 12.8, *SD* = 1.2). The infant's age in months at the visit, which was positively associated with the sociodemographic risk index, *r*(271) = .15, *p* < .05, was included as a covariate in analyses.

### Participants

#### The 1‐Year Lab Visit (Wave 3)

At Wave 3, 312 (92%) of the original 332 families remained enrolled in the study, 291 of whom (93%) were assessed, with 275 able to visit the laboratory and 16 providing questionnaires only. Nine families were unable to be assessed during the time window, due to work commitments, poor health, or adverse family circumstances, and 12 booked appointments but canceled and were unable to reschedule. Four children who participated in individual assessments could not be observed during a birthday party due to other families’ cancellations. The demographics of the subsample of the 271 families that attended the birthday party did not differ significantly from the sample as a whole.

#### 2.5‐Year Lab Visit (Wave 5)

At Wave 5, 309 families remained in the sample; 272 (88%) were assessed at this wave, with 236 (87%) visiting the laboratory and 36 only able to complete questionnaires. Of the 236 who visited the laboratory, 225 (95%) took part in the birthday party. Because of other families’ cancellations, the remaining 5% did not have the opportunity to participate in a birthday party, and thus took part in individual assessments only. Of the 271 families who had participated in the 1‐year party, 225 (79%) were able to come to the laboratory again; they had significantly lower scores on the sociodemographic risk index than did the sample as a whole, *t*(224) = −3.19, *p* < .01.

### Procedure

#### 1‐Year Lab Visit (Wave 3)

Each visit was scheduled at 2 p.m. and lasted approximately 1.5 hr. The timing of the visit and its length were appropriate for a child's birthday party; scheduling the appointments for the afternoon standardized the time of day at which physiological measures were taken (see Hay et al., 2016). The afternoon began with each family being assessed in a separate testing room. The individual assessments included a joint attention task that is described below. The parent and child (along with any accompanying family members) were then escorted to the large playroom for the party.

The party comprised an emotion regulation challenge followed by a free play session. For the emotion regulation challenge, which was designed to resemble the type of dramatic entertainment that often features at young children's birthday parties in the United Kingdom, a researcher joined the party dressed as a “Birthday Lady” in a princess costume. She administered the “Teddy Bear's Picnic” procedure, during which a second researcher dressed in a teddy bear costume entered the room and joined the party, pretended to drink tea and eat toy food, and invited the parents and infants to dance before saying goodbye and leaving the room (see Hay et al., 2016 for more details).

After the Teddy Bear left the room, the families were asked to proceed as they normally would at a child's birthday party with other families, and the infants were observed for 20 min of free play. Two wall‐mounted cameras were used to make video records. At the end of the party, the infants were invited to play Lucky Dip and select a gift‐wrapped book hidden in a box of balls. A £20 gift voucher was given to each family.

#### 2.5‐Year Lab Visit

The procedure for the laboratory visit was replicated exactly when the children were between 30 and 36 months of age (*M* = 33.6, *SD* = 2.5). Again, visits were scheduled for 2 p.m. and lasted approximately 1.5 hr. The individual assessments were changed to be age appropriate. Age‐appropriate toys were provided in the playroom, but the procedures for the Teddy Bear's Picnic and the peer interaction session were replicated exactly. No attempt was made to pair families with the ones observed together at the earlier visit.

The children first took part in individual assessments, which included self‐regulation and imitation tasks, in separate testing rooms, accompanied by their caregivers. Following the individual assessments, families were observed in the playroom during the birthday party, which again comprised the Teddy Bear's Picnic challenge, followed by 20 min of free play.

### Measures

#### The PICS

As in the 1.5‐year home visit, the observational measures of the children's prosocial behavior and aggression were derived from the PICS. At the 1‐year lab visit, independent observers attained significant agreement, *ICC* = 0.80 for *tugging* on toys, *ICC* = 0.79 for *bodily force*, and *ICC* = 0.90 for *offering objects* to a peer. The comparable agreement statistics at the 2.5‐year lab visit were *ICC* = 0.94 for *tugging* on toys and *ICC* = 0.98 for *offering* objects. However, at that age, bodily force occurred so rarely that all possible instances were reviewed and agreed upon by two observers. Inspection of the distributions revealed that they were skewed and kurtotic; a square root transformation was applied to each scale score, which improved the normality of the distributions and permitted the use of parametric statistics.

#### Joint Attention

At the 12‐month visit, joint attention was measured and other age‐appropriate tasks were given during individual testing. The order of assessments was randomized across the participants. The infant sat on the caregiver's lap, across a table from the experimenter. A video camera was placed in one corner of the room approximately 1–1.5 m away from the infant. The testing room was decorated with four brightly colored posters, each of different cartoon characters.

The joint attention task was derived from the Early Social Communication Scales (Mundy et al., 2003), which consisted of four trials. During each trial, the experimenter ensured that the infant was looking at him or her before gazing at and pointing toward one of the posters. The order in which the experimenter pointed to each poster was counterbalanced across participants. The experimenter pointed with his or her index finger while holding the arm next to the torso. During each trial, the experimenter called out the infant's name three consecutive times before moving on to draw attention to the next poster. Data from the joint attention task were available for 258 (94%) of the 275 infants who visited the laboratory at 1 year (for more details on the procedure see Roberts et al., 2013).

Video records of the infant's response to the joint attention task was scored using frame by frame observation to determine where the infant was looking, whether the infant was pointing, and in which direction (i.e., to which poster, or to another location in the room). This enabled precise measurement of the infant's *gaze following* of the experimenter's gaze and point, *gaze alternation* between the target poster and experimenter, and *pointing* during the task. Independent observers scored 16% of video records, identifying gaze following, gaze alternation, and pointing, with good agreement (*κ* coefficients ranged from 0.92 to 0.95). Insofar as the three elements of joint attention were intercorrelated, a principal components analysis yielded a single factor score that accounted for 68% of the variance in joint attention behaviors.

#### Self‐Regulation in Early Childhood

During the individual assessments at the 2.5‐year visit, the children were again presented with a battery of four *self‐regulation* tasks, given in several random orders. All tasks required the child to inhibit a prepotent response: the *Tower of Cardiff* planning task; the *Raisin Task*, a delay of gratification challenge; the *Whisper Task*, an inhibitory control task; and the *Big Bear, Little Bear Task*. a nonverbal Stroop task.

During the *Tower of Cardiff* task, the child was presented with a stacking toy with a plastic pillar and plastic rings of various sizes; the pillar was narrower at the top than at the base, so that rings could be stacked in a graduated order. The experimenter presented the toy with an unusual order of rings and asked the child to copy that order on an empty pillar. Children were given two trials. Their responses were scored as 0 if no tower was built at all; 1 if the tower did not resemble the experimenter's tower and was not the conventional graduated tower; 2 if the child stacked the rings in the graduated order; and 3 if the child copied the experimenter's tower exactly. Reliability was assessed on 57 infants (25%); *ICC* = 1.00.

The *Raisin Task* was adapted from the original “Snack Delay” task in which a child was required to wait to retrieve a colorful candy from under a see‐through cup (Kochanska et al., 1996). In the current work, the researcher placed a raisin underneath a plastic box and instructed the child not to touch or eat the raisin until the researcher rang a bell. The child's response for each of three trials was scored as 0 if the child ate the raisin before the experimenter rang the bell; 1 if the child touched the bell, box, or raisin, but did not eat the raisin; and 2 if the child neither ate the raisin nor touched the bell, box, or raisin during the trial, *ICC* = 0.96. Thus, higher scores indicated greater self‐regulation.

The *Whisper Task* was adapted from a similar task used by Kochanska et al. (1996). Children were presented with a toy farmyard, which was made up of a large plywood base, painted as a yard with a pond and vegetable patch. The experimenter instructed the child to “wake up” 10 plastic farm animals by naming each animal in turn and whispering “good morning” very softly to them. The child's response to each toy animal could be coded as “shout,” “normal voice,” “low vocal sound,” or “whisper,” which were scored as 0, 1, 2, or 3 respectively, *ICC* = 0.98.

The *Big Bear, Little Bear* task was adapted from the baby Stroop task (Hughes & Ensor, 2005). Children were presented with a large, laminated picture of a large bear and a small bear, which was placed on the table. Two spoons (a big spoon and a small spoon) as well as two cups (a big cup and a small cup) were also presented. The experimenter showed the child the picture and explained that Big Bear liked to use a small spoon and a small cup, while Little Bear preferred a big spoon and a big cup. The child was then asked to place the four items on top of the correct bear in the picture during four trials. Children's responses could be coded as “no response,” “conventional response” (e.g., with Big Bear getting the large utensils, which was incorrect) or “correct response.” The number of correct responses across the four trials thus could range between 0 and 4, *ICC* = 0.99.

Because the tasks required children to get information from the experimenter's modeling or instructions, their social learning abilities were controlled for in a factor analysis using Mplus 7, which was run on the scores from each of the four self‐regulation tasks plus two additional imitation tasks in the battery. The factor analysis yielded three factors: *imitation* (the control tasks), *behavioral regulation* (the Raisin Task and Whisper Task), and *cognitive flexibility* (the Tower of Cardiff and Big Bear, Little Bear Task). In a follow‐up analysis, the imitation factor was constrained to be orthogonal to the behavioral regulation factor, which yielded a better fit; the behavioral regulation and cognitive flexibility factor scores were used in the following analyses of the 231 children who took part in laboratory tasks.

## Results

### Preliminary Data Analyses

#### Controls for Group Membership

When measuring the children's sharing and use of force during the laboratory birthday parties and testing for gender differences, it was important to control for the particular combination of participants; the grouping of particular peers with individual aggressive and prosocial tendencies was likely to influence the group dynamics at each party. We used the Linear Mixed Models module on SPSS to analyze the contribution of the particular combination of peers on the measures of observed prosocial behavior and aggression.

#### Checks on the Influence of Group Size

Because some families canceled and rescheduled their visits, only 59% of participants at the 1‐year visit were observed in groups of three, with 32% tested in dyads and 8% in quartets. (A quartet was formed when only one family had arrived at the laboratory at the scheduled time and was added to a later party.) Linear mixed models that controlled for group membership revealed a trend for infants to share more often when they were interacting in dyads (*M* = 3.91, *SD* = 5.66) rather than in triads or quartets (*M* = 2.98, *SD* = 5.89), *F*(2, 83) = 2.58, *p* < .10. Group size did not influence tugging on toys or use of bodily force.

At the 2.5‐year visit, 56% of participants were observed in triads, as planned, with the remaining 44% observed in dyads because of scheduling issues as before. There were no significant effects of group size on the children's peer‐directed behavior at Wave 5.

### Sharing and the Use of Force at the 1‐Year Lab Visit

#### Peer‐Directed Actions

Means and standard deviations for offering objects, tugging on peers’ toys, and deploying bodily force against the peer are shown in Table 7. At the 1‐year birthday parties, half of the sample (50%) offered or gave objects to their peers; 43% tugged on their peers’ toys; and 20% used bodily force against their peers. Much of the bodily force was pushing or pulling on the peers; only 6% of infants hit or tried to bite their peers.

**Table 7:** Means and Standard Deviations of Prosocial Behavior and Use of Force Scores at the 1‐ and 2.5‐Year Laboratory Visits

		Girls		Boys	
Visit	Behavior	Mean	*SD*	Mean	*SD*
1‐year visit	Offers toys	4.07	6.82	2.74	4.71
	Tugs on toys	2.15	3.68	2.47	4.39
	Bodily force	0.93	2.69	1.34	2.91
2.5‐year visit	Offers toys	2.84	4.84	1.98	3.86
	Tugs on toys	2.01	7.50	1.90	5.48
	Bodily force	0.27	2.46	0.17	0.72

Analyses of scores (following square root transformations) revealed that, at 1 year, all three types of social actions were significantly and positively correlated (offering and tugging: *r* = .26, *p* < .001; tugging and bodily force: *r* = .20, *p* < .001; and offering and bodily force: *r* = .15, *p* < .05). A principal components analysis yielded a single dimension, which can be labeled *social engagement*. The social engagement factor accounted for 47% of the variance in the infants’ peer‐directed actions.

#### Associations With Age

The infants’ chronological age was not significantly correlated with the use of force against peers (bodily force: *r* = −.02; tugging on toys: *r* = .04). However, sharing with peers was marginally associated with age (*r* = .12, *p* < .10).

#### Gender Differences

Tests for gender differences took into account the influence of the infants being paired with particular members of the sample. After controlling for group membership, the analyses showed that girls shared more than boys (Table 7), *F*(1, 257.3) = 4.72, *p* < .05. There were no significant gender differences in tugging on toys or using bodily force.

#### Associations With Joint Attention Skills

The joint attention factor score was only marginally associated with the general sociability factor score (*r* = .12, *p* < .10) but significantly correlated with sharing with peers (*r* = .15, *p* < .05).

#### Associations With Parents’ Prosocial and Antisocial Behavior

The infants’ peer‐directed behaviors were not associated with mothers’ or fathers’ history of antisocial behavior. However, infants whose mothers reported higher scores on the sociodemographic risk index were more likely to tug on peers’ toys (*r* = .14, *p* < .05).

### Sharing and the Use of Force in Early Childhood

Means and standard deviations for the PICS scale scores are shown in Table 7. At the 2.5‐year lab visit, 48% of the children offered objects to their unfamiliar peers; 34% tugged on peers’ toys; but only ten children (4%) ever used bodily force. Offering and tugging toys were once again positively correlated (*r* = .43, *p* < .001). Because only ten children used bodily force, a nonparametric analysis was used. Tugging, but not offering, was positively associated with the use of bodily force (*ρ* = .24, *p* < .001). However, when tugging and bodily force were summed to create a single measure of physical aggression, that summary variable was significantly and positively associated with offering objects to the peers (*r* = .22, *p* < .01).

#### Associations With Age

The child's chronological age in months was not associated with peer‐directed behavior at the 2.5‐year visit. No significant associations were found between age in months and tugging on peers’ toys, using bodily force, or offering objects.

#### Gender Differences

After controlling for the effects of group membership, no significant gender differences were seen in sharing, tugging on peers’ toys, or the composite measure of all physical force used against the peers.

#### Associations With the Self‐Regulation Tasks

The children's peer‐directed behaviors were not significantly correlated with their measured self‐regulation skills; the correlations with the behavior regulation and cognitive flexibility factors both approached zero. However, for the 2.5‐year‐olds, offering objects to peers was significantly associated with the social learning factor derived from the control imitation tasks (*r* = .13, *p* < .05).

#### Associations With Parents’ Prosocial and Antisocial Behavior

The children's peer‐directed behaviors were not associated with the sociodemographic adversity index or with the parents’ reports of prosocial behavior, but the children's overall use of physical force against peers was positively associated with fathers’ reports of their own antisocial tendencies (*r* = .18, *p* < .05).

### Changes Between the 1‐ and 2.5‐Year Visits

Because so few children used bodily force at the second laboratory visit, the two types of force (tugging on toys and bodily force) were combined. The total use of physical aggression declined significantly from 1 to 2.5 years (respectively, *M* = 3.48, *SD* = 5.41 vs. *M* = 2.16, *SD* = 7.82); multivariate *F*(1, 207) = 12.33, *p* < .001, partial *η* ^2^ = .06. There was no significant effect of family adversity, and the decrease in the use of force was not moderated by gender.

In contrast, the rate of sharing with peers did not change significantly from 1 to 2.5 years, but there was a significant main effect of gender, *F*(1, 207) = 7.02, *p* < .05, partial *η* ^2^ = .03, with girls more likely than boys to offer objects to peers (see Table 7).

### Stability From 1 to 2.5 Years

There was little evidence for stable individual differences from 1 to 2.5 years for either prosocial behavior or aggression. Sharing at 1 year was only marginally correlated with sharing at 2.5 years (*r* = −.13, *p* < .10). However, against expectations, sharing at 1 year significantly predicted the summary measure of physical force at 2.5 years (*r* = .14, *p* < .05). This pattern might reflect individual differences in general sociability that endured over time. However, the social engagement factor did not show such stability over time.

### Effects of Being Paired With Particular Peers

At each wave of data collection, the composition of the dyads or triads exerted an effect on individual children's social behavior. Linear mixed models were used to control for the effects of group membership (in the dyad or triad) on the children's peer‐directed behavior at each wave.

At 1 year, group membership was significantly associated with sharing (Wald *z* = 2.02, *p* < .05) but not physical aggression. At 2.5 years, the reverse was true: Group membership was significantly associated with children's use of physical force (Wald *z* = 4.32, *p* < .001), but not sharing.

### Associations Between Observed Behavior and Informants’ Ratings

The prosocial factor scores derived from informants’ ratings at the 2.5‐ to 3‐year questionnaire assessment were not correlated with observed sharing. The aggressive factor scores were only marginally associated with the combined measure of tugging and bodily force against peers (*r* = .11, *p* < .10).

## Discussion

The laboratory observations showed that physical aggression mainly took the form of tugging objects away from peers; actual bodily force declined from 1 to 2.5 years. Individual differences were apparent. No gender differences in physical aggression were apparent at either age, but at 1 year, girls offered toys to their peers significantly more often than did boys.

By the time of the 2.5‐year lab visit, families who were observed in the laboratory had lower scores on the sociodemographic risk index. This is a limitation on sample representativeness, which may have reduced variability in the children's behaviors. Nevertheless, the smaller sample size was still sufficient to detect small effects and so the low prevalence of bodily force at 2.5 years was probably not due to lack of statistical power.

The significant dependencies in the data identified in the analyses provided evidence for peer influence that affected sharing at 1 year and aggression at 2.5 years. This finding suggests that, when very young children move into preschool settings, their social interactions are increasingly likely to be influenced by the dynamics of the peer groups in which they find themselves. Their use of prosocial behavior and aggression reflects not only their own skills and their parents’ socializing influences, but also the social environments in which they find themselves. To address these issues further, the next chapter focuses on the children's later decision‐making skills with respect to interactions with peers.

## VI Social Behavior at 7 Years: Cognitive Tasks and Teachers’ Reports

The previous analyses demonstrated that individual differences in prosocial behavior and aggression emerged in infancy and persisted to age 3 years. At the 7‐year visit (Wave 6), we asked, did those individual differences persist into primary school? Were the angry and aggressive children more prone to conflict with classmates? Did the prosocial children adapt more easily to the demands of the school environment? We used experimental tasks to examine cognitive and emotional processes that predicted children's prosocial and aggressive decision‐making in response to peer conflict.

Developmental theorists have drawn attention to social information‐processing in childhood, particularly in the context of conflicts with other children (e.g., Dodge & Crick, 1990; Dodge et al., 1986). In social situations in which it is necessary to make quick decisions, children must try to comprehend what their peers intend and then decide how to pursue their own goals. They may engage in aggression or find ways to avoid conflict, perhaps by acceding to peers’ demands (e.g., Dodge & Crick, 1990). Cognitive and emotional processes underpin such social decision‐making (Lemerise & Arsenio, 2000), which is influenced by children's own self‐concepts and their understanding of gender norms (Ostrov & Godleski, 2010).

Social decision‐making influences both prosocial behavior and aggression (e.g., Carlo et al., 1991). In many everyday situations, children must decide whether or not to share their resources with their companions (e.g., Flook et al., 2019), or whether to respond to another person's distress (Demetriou & Hay, 2004). Children show their awareness of norms governing sharing and bystander intervention by the preschool years (Caplan & Hay, 1989; Rochat et al., 2009), and perhaps even before (Lucca et al., 2018; Ulber et al., 2015).

At the 7‐year assessment, we examined the children's social decision‐making in two experimental tasks, one a conventional task with a scenario enacted by glove puppets and the second a more immersive video game. In the former puppet conflict task, the children were asked to respond to a possession dispute in which one puppet had seized another's possession. Prior research had shown that performance on this task correlated significantly with children's observed conflict with peers (Hay et al., 1992). In the latter Castell Arth Mawr Adventure Game (CAMGAME; Hay et al., 2018), children were given the opportunity to explore a virtual environment in which they encountered provocations from peers and opportunities to help others.

## Associations With Children's Cognitive and Emotional Skills

We expected that children's responses to the decision‐making tasks would be influenced by situational characteristics such as their mood at the time of testing, as well as by their standing tendencies to engage in prosocial behavior and aggression. Social decision‐making also draws upon broader cognitive skills and emotion understanding. In particular, we anticipated that performance on the decision‐making tasks would reflect the child's ability to understand the instructions and recall the details of scenarios presented in the tasks, and therefore decision‐making would be associated with verbal ability and working memory.

We also hypothesized that prosocial decision‐making would be associated with emotion understanding and social cognition. The ability to recognize and interpret emotional cues and understand the inner states of other people contributes to prosocial development (e.g., Imuta et al., 2016). Children with behavior problems sometimes show compromised ability to recognize emotions, which may affect their ability to navigate social situations (Fairchild et al., 2009). A child who relies on aggressive strategies may have problems in other domains of social cognition, such as emotional perspective‐taking (de la Osa et al., 2016) and interpreting the thoughts of others (Hughes et al., 1998; see also Sharp et al., 2007). Thus, in this study, it was important to identify correlates of social decision‐making at age 7.

## Association With Children's Classroom Behaviors

The social decision‐making tasks were designed to present challenges that (a) were similar to the challenges children might face in their daily lives and (b) presented the possibility of responding with either prosocial behavior or aggression. We expected that children's decision‐making would be correlated with how they behaved toward their classmates, as assessed by classroom teachers’ reports of the children's typical behavior at school.

## Method

### Design

The 7‐year assessment (Wave 6) comprised two home visits (to be referred to as Session 1 and Session 2), in which one researcher interviewed primary caregivers about family circumstances, the child's mental health, and their own mental health. The caregivers also completed questionnaires during the visit. A second researcher assessed the child's cognitive and language skills, emotional understanding, and prosocial and aggressive decision‐making on the experimental tasks. In addition, and if parents permitted, classroom teachers completed questionnaires.

### Participants

Of the 321 families remaining in the CCDS sample after the child was born, 286 (89%) participated at 7 years (Wave 6). Of those families, 271 (95%) were observed at home and 16 completed only questionnaires.

At the first Wave 6 session, the mean age of participating children was 6.9 years (*SD* = 0.4); at the second session, mean age was 7.0 years (*SD* = 0.04). In the United Kingdom, children enter the first year of primary school at age 5 and thus participants were not in transition to formal schooling at the time of the Wave 6 assessments.

### Procedure

At each session, one researcher interviewed the primary caregiver (98% mothers) while another researcher assessed the child. When necessary, a third researcher looked after younger siblings. At the end of the session, parents and children were observed during interactive games. For children whose parents had granted permission, a researcher contacted the child's school and obtained questionnaire responses from the classroom teacher. Teachers’ reports were thus obtained for 253 children (88% of the families that were participating at 7 years).

### Social Decision‐making Tasks

As described in more detail below, two experimental tasks were included in the battery of assessments to measure prosocial and aggressive decision‐making. Both decision‐making tasks measured the child's responses to social dilemmas that might provoke conflict with peers.

#### The Peer Conflict Puppet Task (Hay et al., 1992)

Glove puppets (a lion and a tiger) were used to enact a scenario in which one child brought a new possession to school and showed it to a peer. The children were asked what each character might do, what they themselves might do in that situation, and what might be wrong to do (Table 8).

**Table 8:** Protocol for the Puppet Conflict Task at the 7‐Year Home Visit

Now I want to show you a puppet show. This is Lion and this is Tiger. They go to a school for zoo animals. They are in the same class. It is Monday morning. Lion's birthday was yesterday and his grandparents gave him this ball. Tiger loves balls. He tries to grab it.
*(Act out the grabbing of the ball; end with the owner still holding the ball, the other puppet still reaching out for it.)*
1. What does Tiger *(the aggressor)* want?
2. What does Lion *(the owner)* want?
3. Show me how Tiger feels. *(Use pictures)*
4. Show me how Lion feels. *(Use pictures)*
5. Is this a fight?
6. Why *(or why not?)*
7. Whose fault was it? *(If child doesn't answer, say*, Point to the one whose fault it was.)
8. Who is going to win?
9. Who is going to lose?
10. If Tiger wants the ball, what should Tiger do?
11. What would you do?
12. Is there anything Tiger could do that would be wrong?
13. If Lion wants to keep holding the ball, what should Lion do?
14. What would you do?
15. Is there anything that Lion could do that would be wrong?
16. If they were real children, who would you like to play with? *(Ask child to point.)*
17. Show me what happens next. *(Reenact the situation with the puppets and then give the puppets to the child to put on. Repeat, show me what happens next.)*

*Note*. The story reproduced above describes Tiger as the aggressor. However, the assignment of Lion or Tiger as the aggressor was counterbalanced across participants.

The puppet conflict task yields two summary measures including first, *socialized choices* (polite requests or sharing) and second, *aggressive choices* (instrumental aggression or bodily force). Each was scored in response to five of the open‐ended questions. Answers that did not indicate either socialized or aggressive choices were given a score of 0. *Socialized choices* included polite pursuit of self‐interest (e.g., “ask nicely,” “say please”) were given a score of 1; explicitly prosocial choices (e.g., sharing) were given a score of 2, allowing a maximum score of 10 across the five questions. Analogously, *aggressive choices* that mentioned instrumental aggression (e.g., “Grab the ball”) were scored 1, and choices recommending bodily force (e.g., “Punch the lion!”) were scored 2, again allowing a maximum score of 10. Children's aggressive choices on the puppet conflict task had previously been shown to correlate significantly with the duration of their observed conflict with peers (Hay et al., 1992).

#### The CAMGAME (Hay et al., 2018)

The *CAMGAME* is a first‐person perspective game inspired by the classic Robbers Cave experiment (Sherif et al., 1949) in which the experimenters induced conflict between two groups of children attending a summer camp. CAMGAME similarly offered opportunities for conflict with a group of unfamiliar children. The script was then instantiated in a modified version of the game *The Elder Scrolls V: Skyrim* (Bethesda, 2011, 2012), using freely available development tools for that game. The child was given a game controller to use (for details of the game script, see Hay et al., 2018). A clip from CAMGAME is available here: https://youtu.be/SpixvsHypg8.

Scoring criteria for the children's choices in response to aggressive and prosocial challenges embedded in the game are presented in Table 9. During testing, researchers recorded whether an aggressive or prosocial choice was present (scored 1) or absent (scored 0) in response to each challenge. Scores across the challenges were summed and divided by the number of challenges encountered, yielding two proportions, one each for aggressive and prosocial behaviors. Arcsine transformations of the two proportions were computed.

**Table 9:** Scoring Criteria for Children's Responses to Aggressive and Prosocial Challenges in the Castell Arth Mawr Adventure Game at the 7‐Year Visit (Hay et al., 2018)

Event	Vignette	Aggressive choices	Prosocial choices
First push scene	In the distance, in front of the door leading to the next scene, there is a child from another school. As X walks up the path, X's friends from the red school say, “Who's that?” and “He's from that blue school!” When X approaches, the blue school child says, “You red school loser!” and X is pushed away.	X uses the mallet to hit the blue school child	N/A
Blacksmith scene	X enters the scene and walks toward a blacksmith, standing next to an anvil and workbench, who says, “Hello there, I'm the castle blacksmith, could you help me? I see you have a mallet there, could you hit this piece of armor a few times? It's for a game later. To get your mallet out, press the purple button. To put it away press the yellow button.”	N/A	X uses the mallet to hit the armor to help the blacksmith
Storyteller scene	X enters the scene and walks toward an elderly character, the Storyteller, who says, “Hello children I am the Storyteller! This was the castle of the Bear King, before he left the buried treasure in the caves! If you're quick you might find it, before those blue school kids do.”	X uses the mallet to hit the Storyteller	
Storyteller scene woodpile	As X proceeds through this scene to the door, the Storyteller says she is cold and asks, “Before you go, could you help me? Could you hit that woodpile with your mallet? I need it for later to warm up.”		X uses the mallet to hit the woodpile to help the Storyteller build a fire
Cave scene	X enters the scene and is pushed back by the blue school. children, who say, “You red school loser!” and “I pushed you that means go away!”	X uses the mallet to hit the blue school child	X walks over to the injured red school friend
	X's friends from the red school are on the other side of the room. One of the children is on the floor and says, “Ow! They pushed me!”		
	If X approaches the blue school children, X is further taunted, “Yeah we pushed you and your friends, so what? We're gonna get the treasure before you.”		
	If X approaches the red school friend, X hears, “Oh thank you, I'll be okay.”		
Ditch scene	X walks along the path and hears the red school friends say, “Those mean bullies pushed us down here.”	X uses the mallet to hit the blue school child	X walks to injured red school friend
	The blue school children are at the end of the path whilst the red school friends are at the bottom of a staircase. As X moves closer to the blue school children they say, “Oh look, it's stupid again.”		
Racing to the treasure scene	In this final scene, X is trying to find the entrance to the castle. X encounters the red school friends and the blue school children who are also “racing” to the entrance. There is no taunting speech from the blue school children in this scene.	X uses the mallet to hit the blue school child	N/A

### Verbal Ability, Working Memory, Social Cognition and Emotion Understanding

#### Verbal Ability

Each child's vocabulary knowledge was assessed using the British Picture Vocabulary Scale (BPVS; Dunn et al., 1982). Verbal IQ was calculated by age‐normalizing the data to produce a standardized score.

#### Working Memory

The measure of working memory was the Visuospatial Sequencing (VSS) task from an executive function battery, the Amsterdam Neuropsychological Tasks (ANT; de Sonneville, 1999). The ANT is a well‐validated and sensitive instrument that has been used in population‐based samples (Brunnekreef et al., 2007) as well as clinical samples (Rommelse et al., 2008). The tasks were presented on a laptop and children made responses using a mouse. For each task, the experimenter gave instructions whilst showing examples. Children were given a practice trial before the test trials.

In the VSS task, the child was presented with a gray square containing nine circles symmetrically positioned in a 3 × 3 matrix on the screen. After a beep, a sequence of circles was pointed at by a computer‐animated hand. The child was instructed to use the mouse to replicate the sequence over 24 trials which gradually increased in difficulty, with a greater number of target circles and more complex sequences. The VSS score was operationally defined as the total number of correct circles selected in the correct order, out of a possible 100.

#### Second‐Order False‐Belief Task

Children's social understanding was assessed by an age‐appropriate second‐order false‐belief task (Paine et al., 2018) adapted from established second‐order belief paradigms (Coull et al., 2006; Perner & Wimmer, 1985). The experimenter told a story that was enacted with plastic Playmobil® figures. In the story, the protagonist was gender‐matched to the participant and a sibling character was gender‐matched to the participant's closest‐in‐age younger sibling. In cases in which the child had no siblings, the sibling character's gender was randomly selected.

Children were classified as passing second‐order false belief if they answered the first location question correctly and gave an appropriate justification, and as passing second‐order false belief with full comprehension if they answered the additional probe questions correctly. An independent observer coded 33% of the transcripts and established agreement for passing second‐order false belief (*κ* = 1.00) and for appropriate versus inappropriate justifications (*κ* = 1.00). There was also good agreement within appropriate and inappropriate justification codes (*κ* = 0.89 and 0.79, respectively).

#### Emotion Recognition and Perspective‐Taking

Two established tasks were used to measure emotion recognition and emotional perspective‐taking (Denham, 1986; Lane et al., 2010). First, to assess basic emotion recognition, children were asked to match eight faces printed on a card to four target emotions (Van der Schalk et al., 2011). The experimenter asked, “Can you find me someone who is [*scared/cross/happy/sad*]?” Two of each of these four questions were posed. Each correct match between a label and a facial expression was scored as 1, yielding total scores ranging between 0 and 8.

Next, children were given an adapted version of an emotional perspective‐taking task (Pollak et al., 2000). The researcher gave the child a new card that showed four emotions from the previous task, depicted by faces that were gender‐matched to the participant. The researcher then read short vignettes in one of two counterbalanced orders, in which the story protagonist experienced happiness, sadness, anger, or fear (see Table 10). The protagonist was the same gender as the child. Following each vignette, as a memory check, the experimenter asked the child to repeat the story to a puppet and then point at one of the four emotion faces on the card, to indicate how the story protagonist would feel. The child received a score of 1 for every correctly identified emotion per vignette, for a possible total of 8. The scores for the emotion recognition and emotional perspective‐taking tasks were then combined and transformed into to a percentage of correct scores, as a summary measure of emotional *perspective‐taking* (Lane et al., 2010).

**Table 10:** Emotional Perspective‐Taking Task Vignettes at the 7‐Year Visit

*Happiness*
Johnny/Susie wanted his/her friends to come over to play. So he/she asked them, and they came over to play with him/her.
*Sadness*
Johnny's/Susie's friend, who he/she really liked to play with, moved away.
*Fear*
Johnny/Susie was in his/her room at night. It was dark, and he/she saw a tree outside that looked like a person with his hand about to come in the window.
*Anger*
Johnny/Susie let his/her best friend use his/her new ball. His/her friend wasn't careful and lost the ball.
*Fear*
Johnny/Susie was dreaming about a monster in his/her nightmare.
*Sadness*
Johnny/Susie and his/her little sister have a pet dog. The dog is sick and needs to go to the vet's.
*Happiness*
At Christmas, Johnny/Susie got a new toy house that he/she wanted.
*Anger*
Johnny's/Susie's little brother broke his/her favorite toy on purpose.

### Teachers’ Reports of Children's Prosocial Behavior and Aggression

At the 7‐year assessment, 253 classroom teachers completed questionnaires for 88% of the children. The questionnaires included the six‐item CICS (*α* = .82) and a 15‐item age‐appropriate teacher questionnaire that had been developed to measure children's prosocial behavior in classrooms (Weir & Duveen, 1981). Within the CCDS sample, the Weir & Duveen prosocial measure showed good internal consistency, *α* = .94 and test–retest reliability, *r* = .91, *p* < .001.

## Results

### Aggressive and Prosocial Decision‐Making

The descriptive statistics for children's prosocial and aggressive decision‐making scores are presented in Table 11. In response to the five open‐ended questions in the puppet conflict task, 248 children (98%) gave at least one socialized response (polite pursuit of self‐interest or sharing), and 181 (70%) gave at least one aggressive response. In CAMGAME, 251 children (94%) responded in a prosocial way to at least one challenge and 145 children (55%) responded aggressively to at least one challenge. In both tasks, children's positive choices (socialized or prosocial) were more common than aggressive choices. In the Puppet Task, *t*(256) = 21.05, *p* < .001; for CAMGAME, *t*(265) = 15.26, *p* < .001.

**Table 11:** Descriptive Statistics for Social Decision‐Making and Other Variables of Interest at 7 Years

Measure (Statistic 1, Statistic 2)	Statistic 1	Statistic 2
Puppet conflict task: Socialized profile (*M, SD*)	6.84	3.17
Puppet conflict task: Aggressive profile (*M, SD*)	1.68	1.75
CAMGAME: Prosocial choices (proportion) (*M, SD*)	0.61	0.28
CAMGAME: Aggressive choices (proportion) (*M, SD*)	0.25	0.28
Teachers’ report: Prosocial behavior (*M, SD*)	18.80	7.28
Teachers’ report: Aggressive behavior (*M, SD*)	2.88	5.83
Verbal ability standardized score (*M, SD*)	98.90	12.08
Working memory (*M, SD*)	65.73	18.48
Second‐order false belief (*N*, % pass)	76	29.50
Emotion recognition (*M, SD* of % correct)	94.31	10.78
Emotion perspective‐taking (*M, SD* of % correct)	84.56	17.67

*Note*. CAMGAME = Castell Arth Mawr Adventure Game.

Individual differences in decision‐making were apparent, but different patterns were seen across the two tasks. In the Puppet Task, socialized and aggressive responses were negatively correlated, *r* = −.21, *p* < .001. In contrast, in CAMGAME, prosocial and aggressive choices were positively associated, *r* = .19, *p* < .01.

#### Gender

Significant gender differences were found on the Puppet Task, with boys obtaining significantly higher scores on aggression than did girls, boys: *M* = 1.90, *SD* = 1.79, girls: *M* = 1.40, *SD =* 1.66; *t*(255) = 2.26, *p* = .02. However, girls and boys did not differ significantly in socialized responses.

Similarly, in CAMGAME, girls were less likely than boys to make aggressive choices, girls: *M* = 0.11, *SD* = 0.20; boys: *M* = 0.40, *SD* = 0.38; *t*(241.30) = 7.98, *p* < .001. However, girls were also less likely than boys to make prosocial choices, girls: *M* = 0.68, *SD* = 0.46; boys: *M* = 0.82, *SD* = 0.46; *t*(264) = 2.62, *p* < .01.

#### Consistency Across Tasks

The children's socialized responses to the Puppet Task were significantly associated with their prosocial choices during CAMGAME, *r*(255) = .15, *p* < .05. However, aggressive responses to the Puppet Task were only marginally associated with aggressive choices in CAMGAME, *r*(255) = .12, *p* < .10.

#### Social Decision‐Making in Relation to Language and Memory Skills

Descriptive statistics for verbal ability and working memory are presented in Table 11. Children's verbal ability scores on the BPVS were only marginally associated with their socialized responses to the Puppet Task, *r*(250) = .11, *p* < .10, and not significantly associated with prosocial choices in CAMGAME. In contrast, children's BPVS scores were negatively correlated with aggressive choices on both tasks, *r*(251) = −.18, *p* < .01 for aggressive responses to the Puppet Task and *r*(260) = −.14, *p* < .05 for aggressive choices during CAMGAME.

Working memory was not significantly associated with social decision‐making. Aggressive choices on the Puppet Task were marginally correlated with working memory scores, *r*(240) = .12, *p* < .10, but partial correlation analysis showed that the association was accounted for by verbal ability.

#### Social Decision‐Making in Relation to Theory of Mind and Emotion Understanding

Descriptive statistics for the second‐order theory of mind task and the emotion recognition and perspective‐taking tasks are displayed in Table 11. Theory of mind was significantly associated with emotional perspective‐taking, *r*(258) = .18, *p* < .01, but not with recognition of basic emotions. However, despite that association, social decision‐making was correlated with understanding emotion, not theory of mind.

The pattern of associations with emotional understanding differed across the decision‐making tasks. In the Puppet Task, socialized responses were not related to emotional understanding, but children with poorer emotional perspective‐taking were more likely to recommend that the puppets use aggression, *r*(251) = −.25, *p* < .001. In contrast, in CAMGAME, prosocial choices were positively associated with emotion recognition, *r*(265) = .13, *p* < .05, and emotional perspective‐taking, *r*(261) = .19, *p* < .01. Aggressive choices were unrelated to either measure.

### Associations With Teachers’ Reports of Prosocial Behavior and Aggression in School

#### Individual Differences and Gender Differences

Individual differences were apparent for both behaviors, as measured by the 6‐item CICS angry aggressiveness scale, which ranged from 0 to 12 (see Chapter II) and the 15‐item Weir and Duveen (1981) prosocial scale, which ranged from 0 to 30. According to teachers, the CICS and the prosocial scales were inversely correlated, *r*(246) = −.43, *p* < .001.

The teachers rated girls as significantly more prosocial than boys, girls: *M* = 20.92, *SD* = 6.44; boys: *M* = 17.16, *SD* = 7.49; *t*(240.98) = 4.22, *p* < .001, and boys showed more aggression than girls, boys: *M* = 0.91, *SD* = 1.88; girls: *M* = 0.31, *SD* = 0.86; *t*(206.83) = 3.41, *p* < .001.

#### Associations Between Social Decision‐Making and Classroom Behavior

Teachers’ ratings of aggressiveness were positively correlated with the children's aggressive choices during CAMGAME, *r*(244) = .20, *p* < .01. Furthermore, teachers’ ratings of prosocial behavior were inversely correlated with aggressive choices during CAMGAME, *r*(240) = −.15, *p* < .05 and the Puppet Task, *r*(232) = −.15, *p* < .05.

#### Associations Between Teachers’ Ratings and the Children's Cognitive Skills and Emotion Understanding

Correlations between teachers’ ratings of prosocial behavior and aggressiveness and the children's cognitive skills and emotion understanding are presented in Table 12. The teachers’ ratings of prosocial behavior and aggression were significantly associated with the children's verbal ability, working memory, and emotion understanding, but not with theory of mind.

**Table 12:** Pearson‐Product Correlations Between Teachers’ Ratings of Children's Prosocial Behavior and Aggressiveness and Children's Scores on Verbal Ability, Working Memory, False Belief Understanding, Emotion Recognition, and Emotional Perspective‐Taking Tasks at the 7‐Year Visit

	Teachers’ Ratings: Prosocial	Teachers’ Ratings: Aggressiveness
Verbal ability	.27***	−.30***
Working memory	.23***	−.24***
Second‐order false belief	.10	−.02
Emotion recognition	.13*	−.10
Emotional perspective‐taking	.16*	−.12†

^†^ *p* < .10.

* *p* < .05.

*** *p* < .001.

## Discussion

The findings demonstrated that at age 7, children's behavioral, cognitive, and emotional development influenced the ways that they approached the challenges of the social world. Prosocial skills and aggressive tendencies were both evident, with substantial individual variation in each behavior.

At 7 years, in contrast to infancy and toddlerhood, gender differences were evident, both with respect to social decision‐making on experimental tasks and prosocial and aggressive behavior at school. This was the first wave of assessment at which traditional gender stereotypes were observed: Girls were more likely than boys to be prosocial, whereas the boys were more likely than girls to engage in aggression.

Individual differences were apparent, both in the decision‐making tasks and the teachers’ reports. However, the pattern of individual differences was somewhat different in the two experimental tasks. In the Puppet Task, aggressive responses were negatively correlated with socialized ones. However, during CAMGAME, aggressive actions were positively correlated with prosocial ones, which might reflect the level of children's engagement in the game. In some ways, this pattern of response to the social challenges embedded in the game is similar to the observed behavior seen earlier at the laboratory birthday parties (Chapter V): Those children who were most engaged in the situation displayed both prosocial and aggressive behaviors. However, children's aggressive approach to the computer game was corroborated by the teachers’ reports of their aggressiveness at school, showing that their aggressive choices were not completely explained by engagement in the game.

The findings draw attention to the importance of children's cognitive and verbal skills, as well as their emotion understanding. The findings suggest that some children might have difficulties in establishing harmonious relationships with peers in the complex social world of primary school. A minority of them may be on a pathway toward serious, clinically significant behavioral problems, a possibility that we examine further in the next chapter.

## VII Anger, Lack of Empathy, and Clinical Problems: Parents’ and Teachers’ Reports

Our longitudinal analyses highlighted the fact that striking individual differences in angry aggressiveness originate in the first year and predict later behavioral problems. Our findings are in line with many other studies that show continuity in aggressiveness over time in representative community samples (e.g., Côté et al., 2006; NICHD Early Child Care Research Network, 2004), and high‐risk samples (e.g., Lorber et al., 2018), particularly in the context of broader family problems (Shaw et al., 2012). In previous work, we reported that children's early angry aggressiveness predicted clinically significant behavioral problems by 7 years (Perra et al., in press). However, past work has revealed little about prosocial behavior in relation to behavioral problems. Is prosocial behavior a protective factor, so that children who are both aggressive and prosocial might have better outcomes than children who are aggressive only? Alternatively, might it be the absence of empathy and prosocial skills, not physical aggressiveness alone, that sets children on the path to serious behavioral disorders?

With respect to the first question, it is not clear that high levels of prosocial behavior are protective. Indeed, there is scant evidence that individual differences in prosocial behavior are stable over time. For example, data from an earlier longitudinal study provided evidence of continuity in sharing from early childhood to young adulthood, but it offered little evidence for continuity in other prosocial behaviors (Eisenberg et al., 1999).

However, whether or not the presence of prosocial behavior is protective, its absence (in particular, a lack of empathy) is a risk factor for later problems. Some children possess *CU* traits, that is, a lack of concern for others and deficient understanding of their own and other people's emotions (e.g., Frick et al., 2014). Such children are not merely aggressive; they may also find it difficult to engage positively with other people. A recent meta‐analysis has shown that, across samples and age groups, CU traits are strongly and inversely related to measures of empathy and a more general prosocial disposition (Waller et al., 2020).

The absence of empathy, coupled with high levels of aggression, characterizes some children whose behavioral problems are so severe that they meet criteria for clinically significant CD and/or oppositional‐defiant disorder (ODD). These two disorders often co‐occur, but ODD is defined by symptoms of anger and disobedience whereas CD is defined by physical aggression and other antisocial behaviors. In the most recent diagnostic manual (DSM‐5), ODD and CD are both included within a broader diagnostic group of behavioral disorders.

Risk factors for CD and ODD include lower socioeconomic status and parents’ own history of behavioral and emotional problems (Loeber & Hay, 1997). This association may reflect some genetic influence (e.g., Dick et al., 2011; Lahey et al., 2011) as well as the family environment. These disorders are often negatively correlated with verbal ability, executive function, social cognition, emotion regulation, and emotion understanding (e.g., Boden et al., 2010; Van Goozen et al., 2007). To explore the possibility that low levels of empathy places children at risk for behavioral disorders, we focus in particular on three dimensions of CU traits (e.g., Frick et al., 2014) which can be seen conceptually as the inverse of prosocial behavior.

We turn next to testing the developmental hypothesis that early individual differences in angry aggressiveness predict later behavioral problems and clinically significant disorders. We also test whether children's cognitive skills and emotional understanding are inversely related to behavioral problems. To explore the possible protective functions of earlier prosocial behavior, we ask whether children who are highly prosocial at young ages are less likely to develop CU traits and clinical disorders later.

## Method

### Participants

Of the 321 families who remained in the CCDS after the birth of the child (97% of those recruited during the pregnancy), 287 (89%) participated at age 7, with 268 (81%) agreeing to take part in the clinical interview.

### Procedure

At Wave 6, the primary caregiver's interview and questionnaire included questions about the child's behavioral and emotional problems A third questionnaire was left in the home for another parent figure to complete. Because only 58% complied with this request, and because returns were not representative of the demographics of the full sample, the analyses in this chapter focus only on questionnaire and interview data from primary caregivers and the teacher questionnaires previously described in Chapter VI.

### Measures

#### The Child's Prosocial Behavior

The parent questionnaire also incorporated the measure of prosocial behavior (Weir & Duveen, 1981). Reliability on the prosocial scale was *α* = .89 for parents and *α* = .94 for teachers. Parents’ and teachers’ reports of the child's prosocial behavior in the two different contexts (home and school) were significantly, but only modestly correlated, *r* = .22, *p* < .01.

#### The Child's Aggressive Behavioral Problems: Questionnaire Measures

The Child Behavior Checklist (CBCL version 1.5–5 years; Achenbach & Rescorla, 2000) was administered to parents, while the comparable Teacher Report Form was administered to teachers (TRF; Achenbach & Rescorla, 2000). The CBCL/TRF was completed by 274 primary caregivers (97% mothers, 2% fathers, and 1% grandmothers) and 251 teachers. Mothers’ reports on the CBCL aggression scale (*M* = 6.66, *SD* = 6.64) were significantly associated with the teachers’ reports on the TRF (*M* = 2.89, *SD* = 5.84, *r* = .50, *p* < .001).

#### CU Traits

The questionnaires completed by parents and teachers also incorporated a measure of CU traits which had been validated in a childhood sample (Frick et al., 2014). A principal components analysis of both teachers’ and principal caregivers’ reports replicated the factor structure found by Frick and colleagues, yielding three orthogonal factors labeled as *uncaring, unemotional*, and *callous* traits. In the present sample, the caregivers’ and teachers’ reports were significantly correlated on all three scales (uncaring traits: *r* = .24, *p* < .001; unemotional traits: *r* = .24, *p* < .001; and callousness: *r* = .27, *p* < .001).

#### Diagnoses of CD and/or ODD

Trained interviewers interviewed the primary caregiver about the child's current mental health and behavioral difficulties, using the Preschool Age Psychiatric Assessment (PAPA; Egger & Angold, 2004). The interview data were analyzed independently by Gordon Keeler at Duke University, with consultation from Adrian Angold, who had advised us to use the preschool age instrument for the 6‐ to 7‐year‐old children being assessed. Their diagnostic algorithms yielded DSM‐IV clinical diagnoses. We used the combined diagnosis of DSM‐IV CD and/or DSM‐IV ODD with evidence for impairment as the clinically significant diagnostic measure of the children's aggressive problems.

## Results

### Children's Prosocial Behavior at the 7‐Year Visit

#### Individual Differences in Social Behavior

Individual differences were apparent for both prosocial behavior and aggression. An inverse association between the CICS scale and the prosocial scale was found in both parents’ and teachers’ reports (parents: *r* = −.34, *p* < .001; teachers: *r* = −.43, *p* < .001).

#### Gender Differences

As reported in the previous chapter, teachers rated girls as significantly more prosocial than boys, girls: *M* = 20.92, *SD* = 6.44; boys: *M* = 17.16, *SD* = 7.49; *t*(240.98) = 4.22, *p* < .001. Parents similarly reported that girls were more prosocial than boys, girls: *M* = 22.23, *SD* = 5.02; boys: *M* = 19.51, *SD* = 5.86, *t*(280) = 4.13, *p* < .001.

### Children's CU Traits

Girls were significantly less likely than boys to show uncaring traits, as measured by the factor scores from parents’ reports, girls: *M* = −0.28, *SD* = 0.92; boys: *M* = 0.20, *SD* = 1.00, *t*(268) = −4.01, *p* < .001 and from teachers’ reports, girls: *M* = −0.17, *SD* = 0.86; boys: *M* = 0.17, *SD* = 0.96, *t*(242.9) = −3.62, *p* < .001. Girls and boys did not differ significantly in unemotional traits or callousness, only in their lack of concern for others. The sociodemographic risk index was modestly though significantly associated with parents’ ratings of unemotional traits (*r* = .14, *p* < .05), teachers’ ratings of uncaring traits (*r* = .27, *p* < .001), and teachers’ ratings of callousness (*r* = .17, *p* < .01). The children's CU traits were negatively correlated with parents’ and teachers’ ratings of prosocial behavior (Table 13). Parents’ reports of CU traits negatively predicted teachers’ reports of prosocial behavior and vice versa.

**Table 13:** Pearson‐Product Correlations Between Parent Ratings (PR) and Teacher Ratings (TR) of Children's (a) Prosocial Behavior and (b) Callous‐Unemotional (CU) Traits at 7 Years

Child's CU Traits Parent or Teacher Ratings	Child's Prosocial Behaviors Parent Ratings	Child's Prosocial Behaviors Teacher Ratings
CU Uncaring: PR	−.57***	−.14*
CU Uncaring: TR	−.19**	−.68***
CU Unemotional: PR	−.41***	−.16*
CU Unemotional: TR	−.03	−.30***
CU Callous: PR	−.24***	−.14*
CU Callous: TR	−.27***	−.39***

* *p* < .05.

** *p* < .01.

*** *p* < .001.

### Aggressive Problems (CBCL/TRF Scales)

#### Gender and the Demographic Risk Index

Parents’ and teachers’ ratings on the CBCL and TRF aggressive problems scores, respectively, were positively correlated (*r* = .50, *p* < .001). To obtain a measure of serious aggressive behavior problems across the contexts of home and school, a composite factor score was created, which drew upon both the parents’ and teachers’ reports, taking into account shared variance with the sociodemographic risk index which was associated with both informants’ reports. A significant gender difference was observed in the aggressive problems score, with girls below and boys above the mean, girls: *M* = −0.72, *SD* = 2.01; boys: *M* = 0.50, *SD* = 3.44; *t*(284) = −3.50, *p* < .001.

#### Associations With Cognitive Skills and Emotional Understanding

Associations between the CBCL/TRF aggressive problems score and the child's performance on cognitive and emotional tasks are presented in Table 14. In a subsequent linear regression analysis, gender was entered at the first step, and the cognitive and emotion understanding tasks entered at the second step. Results showed that the aggressive problems score was significantly predicted by gender, with boys showing more aggression, *β =* .14, *p* < .05; by verbal ability, *β* = −.23, *p* < .001; and by working memory, *β* = −.26, *p* < .001, adjusted *R* ^2^ = .21, *F*(6, 232) = 11.74, *p* < .001.

**Table 14:** Pearson‐Product Correlations for Children's Aggressive Behavior Problems (CBCL/TRF) and Scores on Verbal Ability, Working Memory, Emotion Recognition, Emotional Perspective‐Taking, and False‐Belief Understanding Tasks at the 7‐Year Visit

	CBCL/TRF Aggressive Problems Score	Verbal Ability	Working Memory	Emotion Recognition	Emotional Perspective‐taking	False‐Belief Understanding
CBCL/TRF aggression		−.34***	−.35***	−.16*	−.23***	−.17**
Verbal ability			.32***	.20**	.37***	.24***
Working memory				.25***	.35***	.12†
Emotion recognition					.36***	.11†
Emotional perspective‐taking						.18**
*N*	286	262	252	268	264	258
*M*	−0.03	98.90	65.73	94.31	84.56	0.30
*SD*	2.97	12.08	18.48	10.78	17.67	0.46

*Note*. CBCL = Child Behavior Checklist; TRF = Teacher Report Form (Achenbach & Rescorla, 2000).

^†^ *p* < .10.

* *p* < .05.

** *p* < .01.

*** *p* < .001.

#### Associations With CU Traits

The aggressive problems score was significantly associated with parents’ ratings of the child's callousness (*r* = .39, *p* < .001), and uncaring traits (*r* = .23, *p* < .001). The aggressive problems score was likewise associated with teachers’ ratings of the child's callousness (*r* = .75, *p* < .001) and uncaring traits (*r* = .38, *p* < .001).

In a linear regression analysis, with gender and the sociodemographic risk index entered at the first two steps, and the child's BPVS score and working memory score entered at the third step, the parents’ report of callousness (*β* = .28) and uncaring traits (*β* = .13) contributed significantly to the prediction of the aggressive problem score, *F*(6, 234) = 19.48, *p* < .001, adjusted *R* ^2^ = .32.

When the parents’ ratings of CU traits were replaced by the teachers’ ratings, the contribution of the child's BPVS was no longer significant and the association with working memory was reduced to a nonsignificant trend. Rather, what predicted most of the variance in aggressive problems were the teachers’ ratings of callousness (*β* = .69) and uncaring traits (*β* = .31), as well as the sociodemographic risk index (*β* = .13), *F*(6, 219) = 92.83, *p* < .001, adjusted *R* ^2^ = .71. Thus, in teachers’ reports, the child's aggressive problems were almost completely bound up with a lack of concern for others.

#### Developmental Continuity Over Time

Analysis of informants’ ratings of aggressive problems revealed considerable developmental continuity over time. Aggressive problem scores were significantly predicted by the child's earlier angry aggressiveness, as measured by the multi‐informant CICS scores at infancy (*r* = .15, *p* < .05); at 1.5 years (*r* = .16, *p* < .05); and at 2.5–3 years (*r* = .23, *p* < .001). The CBCL aggressive problems score at 2.5–3 years significantly predicted the combined CBCL/TRF aggressive problems score at 7 years (*r* = .26, *p* < .001). Despite the emergence of gender differences by age 7, continuity in individual differences in aggressive problems was shown by girls (*r* = .33, *p* < .001) as well as boys (*r* = .21, *p* < .05).

### Clinically Significant Outcomes: DSM‐IV Diagnoses of ODD and/or CD

The Psychiatric Assessment for Preschool Age (PAPA) interview with the primary caregiver at the 7‐year home visit yielded DSM‐IV diagnoses of CD and ODD, generated by the diagnostic algorithms used by Egger and Angold (2004). To meet their clinical diagnostic criteria, the interviewers’ questions needed to establish that children displayed the key symptoms and, in addition, showed signs of clinical impairment in their daily lives at home and school. Thus, for the purposes of the current analyses, the measure of clinical outcome is a diagnosis of either CD or ODD, and for either, an accompanying clinical impairment.

Thirty‐three children (12% of those whose parents were interviewed) met the diagnostic criteria. Boys were more than twice as likely than girls to be diagnosed, with 9 girls (8% of girls) and 24 boys (17% of boys) meeting criteria, *χ* ^2^(1) = 4.29, *p* < .05, odds ratio [OR] = 2.31, 95% confidence interval [CI]: 1.03 to 5.18.

#### Associations With CU Traits

The children who met the diagnostic criteria for ODD and/or CD had higher levels of CU traits, as reported by teachers as well as parents. The combined diagnosis was significantly associated with reports of callousness made by both groups of respondents (parents: *r* = .35, *p* < .001; teachers: *r* = .21, *p* < .01). Mothers’ reports of uncaring traits also predicted the clinical diagnoses (*r* = .20, *p* = .001). Teachers’ reports showed a similar but nonsignificant trend (*r* = .11, *p* < .10). Unemotional traits were not associated with the children's diagnoses.

#### Prediction of CD/ODD From Earlier Angry Aggressiveness

**Figure 2:**
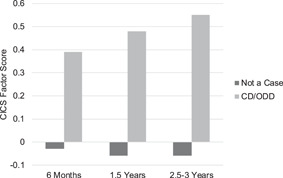
Mean Cardiff Infant Contentiousness Scale (CICS) factor scores for children who did and did not receive conduct disorder (CD) and/or oppositional‐defiant disorder (ODD) diagnoses at 7 years.

In Figure 2, means for CICS scores at 6 months, 1.5 years, and 2.5–3 years are shown for children who did and did not meet the diagnostic criteria for CD/ODD at age 7. The differences between the CD/ODD group and the other children are significant (*p* < .05 or less at each time point), with increasing differences between the groups as the children grew older.

In an initial logistic regression analysis, which included predictors of gender, the sociodemographic risk index, and the mother's own history of antisocial behavior, only the mother's antisocial behavior remained a significant predictor of the child's diagnosis of CD/ODD (*β* = .12, Wald statistic = 7.73, *p* < .001, Nagelkerke *R* ^2^ = .13).

In a second analysis, the mother's history of antisocial behavior was retained as a predictive factor in the model. The cross‐informant factor scores for children's angry aggressiveness and prosocial behavior at the 2.5‐ to 3‐year assessment were added at the second step. There was no evidence for a significant association between earlier prosocial behavior and the diagnosis of CD/ODD in middle childhood. The mother's antisocial behavior remained a significant predictor (*β* = .10, Wald statistic = 5.90, *p* < .05), but in addition, the child's angry aggressiveness at 2.5 to 3 years also predicted the combined diagnosis of CD/ODD (*β* = .81, Wald statistic = 8.30, *p* < .01, Nagelkerke *R* ^2^ = .15).

Was angry aggressiveness predictive at an even earlier age? In a final logistic regression analysis, the measures of angry aggressiveness and prosocial behavior from the 2.5‐ to 3‐year assessment were exchanged for the equivalent measures obtained at the 1.5‐year visit. The mother's antisocial behavior remained a significant predictor of the child's combined diagnosis (*β* = .11, Wald statistic = 6.83, *p* < .05) and again prosocial behavior did not predict the clinical outcome. However, angry aggressiveness at the 1.5‐year visit significantly predicted the child's CD/ODD diagnosis at 7 years (*β* = .62, Wald statistic = 6.04, *p* < .05, Nagelkerke *R* ^2^ = .13).

### Was Early Prosocial Behavior Protective?

The foregoing analyses demonstrated that angry aggressiveness in the early years was a significant predictor of later aggressive behavior problems and, for a minority of children, clinically significant disorders. But could children's early prosocial tendencies protect them against the development of behavior problems? To address this question, we examined the informants’ ratings of prosocial behavior at the 1.5‐year and 2.5‐ to 3‐year assessments and, in addition, children's directly observed prosocial behavior with familiar peers at the 1.5‐year home visit. The outcome variables in middle childhood were the CBCL/TRF aggressive problems score and the parent‐rated and teacher‐rated CU uncaring factors, which can be interpreted as the inverse of prosocial behavior at age 7. Bivariate correlations are presented in Table 15.

**Table 15:** Pearson‐Product Correlations Between Directly Observed Sharing at 1.5 Years, Informant‐Rated Prosocial Behavior at 2.5–3 years, and Later CBCL/TRF Aggressive Problem Scores and CU Uncaring Behaviors as Rated by Parents and Teachers at 7 Years

	1.	2.	3.	4.	5.	6.
1. 1.5‐year observed offering toys						
2. 1.5‐year observed adding toys	.30***					
3. 2.5‐ to 3‐year prosocial factor	.04	.21***	.68***			
4. 7‐year CBCL/TRF aggression score	−.06	−.07	−.07	−.13*		
5. 7‐year CU uncaring: Parent rating	−.16*	−.05	−.23***	−.30***	.23***	
6. 7‐year CU uncaring: Teacher rating	.04	−.15*	−.10	−.06	.38***	.23***

*Note*. CBCL = Child Behavior Checklist; CU = Callous Unemotional; TRF = Teacher Report Form.

* *p* < .05.

*** *p* < .001.

#### Early Prosocial Behavior and the CBCL/TRF Aggressive Problems Score

The parents’ rating of the child's prosocial behavior at 2.5–3 years was negatively correlated with the aggressive problems factor score at age 7 years, but that association was no longer significant when controlling for gender and the sociodemographic risk index. There were no other indications that early prosocial behavior protected children against the risk of aggressive behavior problems later in childhood.

#### Early Prosocial Behavior and Parent‐Rated Uncaring Traits

In contrast, both directly observed and informant‐rated prosocial behavior in the early years were inversely related to CU traits, in particular uncaring traits. The pattern of associations was slightly different for the parents’ and teachers’ reports, but both identified potentially protective functions of early prosocial behavior.

Parents’ reports of the child's uncaring traits at age 7 were negatively associated with their reports of the child's prosocial behavior at 2.5–3 years, and with the child's directly observed sharing with peers at 1.5 years. In an initial linear regression analysis of parent‐rated uncaring traits, with gender (girls coded as 1 and boys as 2) and the sociodemographic risk index entered at the first step, and the 1.5‐year and 2.5‐ to 3‐year prosocial factors entered at the second step, gender was a significant predictor, with boys rated higher on the uncaring trait measure (*β* = .19). Prosocial behavior at 2.5–3 years (*β* = −.23) was inversely related to uncaring traits, *F*(4, 245) = 7.60, *p* < .001, adjusted *R* ^2^ = .11.

The analysis was repeated in the subsample where children had been observed with a familiar peer at the 1.5‐year visit. The focal child's rate of offering objects to the peer was entered at the final step. In the context of the other variables, the observed sharing with the peer was only marginally significant (*β* = −.14, *p* < .10), while being a boy (*β* = .19) and parent‐rated prosocial behavior at 2.5–3 years (*β* = −.23) remained significant predictors of uncaring traits, *F*(5, 167) = 5.22, *p* < .001, adjusted *R* ^2^ = .11.

#### Prosocial Behavior and Teacher‐Rated Uncaring Traits

The predictor variables of gender (again, with girls coded as 1 and boys as 2), the sociodemographic risk index, and the informants’ ratings of prosocial behavior at 1.5 and 2.5–3 years were also used in a regression analysis of teacher‐rated uncaring traits at age 7. Gender (being a boy, *β* = .18) and a higher sociodemographic risk index (*β* = .25) both predicted the teachers’ ratings of uncaring traits, *F*(4, 229) = 7.43, *p* < .001, adjusted *R* ^2^ = .10.

The analysis was repeated for the subsample in which the child had been observed with a familiar peer at 1.5 years, now including *cooperative sharing* (adding toys to the peer's array of play items) as well as *offers to peers* as predictor variables. In this analysis, gender was no longer a significant predictor of the uncaring traits; rather, the sociodemographic risk index (*β* = .25) and observed cooperative sharing (*β* = −.17) were positive and negative predictors of uncaring traits, respectively, *F*(5, 163) = 4.61, *p* = .001, adjusted *R* ^2^ = .10.

## Discussion

By middle childhood, it was clear that the high levels of angry aggressiveness that had first emerged in infancy had consolidated into aggressive behavior problems and, in a minority of cases, problems severe enough to meet criteria for CD and/or ODD. At the same time, individual differences in prosocial behaviors (as well as their inverse, CU traits) had also emerged, as reported by both parents and teachers.

It is noteworthy that by 7 years, gender differences were apparent in both aggression and prosocial behavior. Girls and boys were seen differently in the adult informants’ reports, but those differences are not attributable only to gender biases; the informants’ reports had been validated by observed differences between girls and boys on the social decision‐making tasks. The gender differences in prosocial behavior and aggression that are apparent by 7 years may partly reflect maturation, but they are also likely to reflect social influences that promote gender role socialization, both at home and school. It was particularly striking that, at this point in childhood, boys were more at risk than girls for aggressive behavioral problems and clinically significant diagnoses.

Despite this increasing differentiation between groups of boys and girls, the longitudinal analyses clearly revealed continuity in individual differences over time, highlighting a developmental pathway from angry aggressiveness in infancy toward clinically significant symptoms and disorder. In contrast, prosocial behavior showed less stability over time, perhaps because prosocial actions are more affected by situational factors, often being exhibited in response to the needs of others and events that call for positive actions. Nevertheless, it is noteworthy that early prosocial behavior was an inverse predictor of CU traits, which in turn predicted clinically significant levels of aggressive problems. These findings suggest that it was not just the early presence of anger and aggression but the absence of a concern for other people that placed a minority of the children at risk for serious behavior problems and clinically significant disorders by age 7. This finding further underscores the importance of studying the development of prosocial behavior and aggression in parallel, within the same sample.

## VIII Summary, Limitations, and Implications

We designed our work to study the early development of prosocial behavior and aggression in parallel. To do this, we recruited a nationally representative British sample of over 300 parents who were expecting their first child. We then studied the child's development over 7 years in a two‐construct, multimethod longitudinal design, which revealed developmental trends and the growth of individuality in prosocial behavior and aggression.

Not all infants were aggressive or prosocial. Individual differences were identified in the first 2 years. Early prosocial behavior was not a fleeting, trivial phenomenon, but rather it predicted children's later concern for other people's wellbeing. Angry aggressiveness in infancy also had predictive power and proved to be informative for identifying children who were at greatest risk for later behavior problems. In our closing chapter, we highlight some key findings, acknowledge some of the study's limitations, and reflect on implications for future scholarship and practice.

## Study Highlights

### Prosocial Behavior Was Not Less Common than Aggression

At the 12‐month laboratory visit, infants engaged in early forms of prosocial behavior and aggression. They shared toys more often than they tugged on peers’ toys or used physical force against their peers’ bodies. At the 1.5‐year home visit, when playing with familiar peers, the children shared and tugged on toys at similar rates, and sometimes used bodily force. By the time of the 2.5‐year birthday party, although children still tugged on toys, bodily force was almost never used. Conflict between peers occurred at all ages, but most children who engaged in conflict also shared and took part in cooperative play with their peers.

### Prosocial Behavior and Aggression Become Disentangled Over Time

Although prosocial behavior and aggression could be seen as polar opposites on a single continuum, some observational researchers had drawn attention to positive associations between the two kinds of behaviors in young children (Garner & Dunsmore, 2011; Gill & Calkins, 2003; Green, 1933; Murphy, 1937). Our observations of children's interactions with unfamiliar peers are consistent with that evidence. At both the 12‐month and 2.5‐year birthday parties, children's sharing and the use of force were positively associated. At both ages, principal components analyses yielded a single factor that reflected general sociability and children's willingness to engage with unfamiliar peers. A somewhat different picture emerged when 1.5‐year‐olds played at home with familiar peers: in this context, sharing and aggression occurred at similar rates, but they were not correlated.

By the 7‐year visit, a negative association between prosocial behavior and aggressiveness was found. This pattern was evident from analyses of parents’ and teachers’ ratings of children's behaviors via questionnaires and their decision‐making in the puppet conflict task. In addition, each of these social behaviors showed a distinct pattern of associations with cognitive and emotional skills. The undifferentiated sociability that had been observed at the first birthday party had developed into two distinct styles of relating to peers.

### Early Individual Differences Predict Long‐term Outcomes

Past analyses of data from the larger CCDS from which the current data were drawn had already documented continuity in angry aggressiveness (Hay et al., 2014; Perra et al., in press). Other investigators have also found early appearance of individual differences in aggression (e.g., Alink et al., 2006; Côté et al., 2006; Keenan & Wakschlag, 2002; NICHD Early Child Care Research Network, 2004). However, relatively few researchers have examined the continuity of individual differences in prosocial behavior in studies with longitudinal designs. Our finding of stable individual differences from 1.5 to 3 years corroborates Eisenberg et al.'s (1999) findings of stability in children's sharing. Our data are likewise consistent with Knafo and Plomin's (2006) report of stability in prosocial behavior from 2 to 3 years.

Furthermore, our findings also revealed that early prosocial behavior predicted lower levels of CU traits at 7 years. This finding suggests the potential importance of young children's concern for other people is a foundation for fostering later prosocial behavior and reducing aggression and cruelty.

### Gender Differences in Prosocial Behavior May Precede Gender Differences in Aggression

Earlier analyses of the CCDS data set had shown a univariate correlation between male gender and angry aggressiveness at 6 months of age; however, this association was no longer significant when family risk factors for aggression were taken into account (Hay et al., 2011). In the more extensive analyses reported in this monograph, no gender differences in either directly observed or informant‐rated measures of aggression were found before the age of 7 years.

There were some signs of a gender difference in early prosocial behavior, but the findings were inconsistent. At the 12‐month birthday party, girls were significantly more likely than boys to offer toys to their peers. However, at the 1.5‐year and 2.5‐ to 3‐year assessments, no gender differences were apparent in observed behavior, but at both ages, the informants reported that girls were more prosocial than boys. The disparity between observational and questionnaire data might be due to the broader range of prosocial activities measured in the questionnaires. However, it might also reflect the informants’ gendered expectations for girls versus boys. These mixed findings suggest that gender differences in prosocial behavior begin to emerge in the early years, but context is important.

By the 7‐year visit, gender differences in both prosocial behavior and aggression were evident. Both parents and teachers reported that girls were more caring than boys, and that boys were more aggressive than girls. The informants’ reports of gender differences were corroborated by girls’ and boys’ performance on the social decision‐making tasks.

These longitudinal findings suggest that the difference in girls’ and boys’ social behaviors develops gradually over time. It seems likely that the marked differences at age 7 derived from a developmental cascade of influences. Factors that promote gender differences in social behavior might include girls’ maturational advantage in acquiring language skills sooner (Bornstein et al., 2004); boys’ elevated risk for neurodevelopmental problems (Russell et al., 2014); gender role socialization at home and school (Martin et al., 2002); and children's own roles in the gender segregation of preschool peer groups, leading to what Maccoby (1999) characterized as two separate, gendered worlds of childhood. Cultural expectations for girls versus boys are influential.

Nevertheless, as has been noted in long‐term longitudinal studies (e.g., Moffitt et al., 2001), girls and women continue to show aggression into adulthood, just as boys and men may show generosity and helpfulness throughout life. Our findings similarly suggest that, in the first 3 years, prosocial behavior and aggression both feature in girls’ and boys’ social interactions.

## Study Limitations and the Need for Future Work

Our study had strengths, but also limitations. We recruited a volunteer sample of first‐time mothers who were willing to take part in a longitudinal study. It is important to acknowledge that findings from a study of a moderate‐sized cohort, recruited from a particular city in the United Kingdom, may not generalize to other populations. It was reassuring to learn that the sociodemographic characteristics of the sample were well matched to UK population averages, but there is no doubt that factors linked to the particular participants, time, and place limit the generalization of our findings.

The representativeness of the sample was further reduced by our decision to recruit only first‐time parents. We had aimed to study mothers’ and fathers’ transition to parenthood as the starting point of the study. However, patterns seen for these firstborn children may or may not generalize to later‐born children. Parents’ own child‐rearing strategies might be influenced by their experiences with their firstborns, which would have implications for younger siblings’ behavior. It will be important for future investigators to examine the development of prosocial behavior and aggression in samples that represent different birth orders as well as different sociocultural contexts.

Apart from concerns about the representativeness of the sample, there are two major limitations of our longitudinal design, one at each end of our data collection. The first is that we did not collect data during the preschool years and the second is that we did not collect observations of peer interactions at the final 7‐year visit. With respect to the first, it would have been highly desirable to have observed the children during the preschool years given that this is a time at which children are joining preschool peer groups, when their social cognitive abilities are consolidating, and when gender differences are emerging full force. Clearly these developmental factors could have important impacts on prosocial behavior and aggression. Unfortunately, funding limitations did not permit us to carry out the preschool‐age assessment we would have liked to have included. The absence of direct observation of the focal children interacting with peers at the final 7‐year visit is also a limitation. At the earlier ages, direct observations of peer interaction complemented and validated the informants’ reports, thereby reducing concerns about potential bias in findings from questionnaires. The decision to omit a peer observation at the 7‐year visit was based on judgments about feasibility. In view of the increasing complexity of children's and parents’ lives, expanding involvement in after‐school activities, and other demands on parents’ and children's time, we anticipated that it would be difficult to schedule enough laboratory visits. As seen in Chapter V, the number of participants who could come to the laboratory had already declined between the 1‐ and 2.5‐year visits. As mothers returned to paid employment after the period of maternity leave, they found it more and more difficult to schedule laboratory visits. Likewise, it was difficult to coordinate home visits with another parent and child. In addition, by the time the participant child was 7 years of age, many had younger siblings which made peer observations even more complicated. We thus decided to assess children's general approaches to peer relationships by administering the social decision‐making tasks and by obtaining reports from teachers and parents. Although these data indeed proved to be informative, it will be important for future researchers to expand data collection by conducting behavioral observations at both younger and older ages.

In short, although our work does not allow us to provide definitive answers to all questions about how prosocial and aggressive behaviors develop within individual children, our findings provide a rich foundation for future work, and offer strong support for the value of empirical work that studies—rather than assumes—how these two important categories of social behavior develop within individuals over time.
